# Ethnoracial and social trends in breast cancer staging at diagnosis in Brazil, 2001–14: a case only analysis

**DOI:** 10.1016/S2214-109X(19)30151-2

**Published:** 2019-05-13

**Authors:** Isabel dos-Santos-Silva, Bianca L De Stavola, Nelson L Renna, Mário C Nogueira, Estela M L Aquino, Maria Teresa Bustamante-Teixeira, Gulnar Azevedo e Silva

**Affiliations:** a Department of Non-Communicable Disease Epidemiology, London School of Hygiene & Tropical Medicine, London, UK; b Population, Policy and Practice Programme, University College London Great Ormond Street Institute of Child Health, London, UK; c Departamento de Epidemiologia, Instituto de Medicina Social, Universidade do Estado do Rio de Janeiro, Rio de Janeiro, Brazil; d Departamento de Saúde Coletiva, Faculdade de Medicina, Universidade Federal de Juíz de Fora, Juíz de Fora, Brazil; e Instituto de Saúde Coletiva, Universidade Federal da Bahia, Salvador, Bahia, Brazil

## Abstract

**Background:**

Policies for early detection of breast cancer, including clinical breast examinations and mammographic screening, were introduced in Brazil in 2004, but their effect on disease stage at diagnosis is unclear. We aimed to assess whether these policies have led to a decrease in the prevalence of late-stage breast cancer at diagnosis.

**Methods:**

In this case only analysis, using an anonymised nationwide hospital based-cancer registry network, we identified women aged 18–89 years who had been diagnosed with an invasive breast cancer in Brazil during 2001–14. We extracted individual patient-level data on patient demographics, tumour variables, and health-care provider variables for the centre where the patient was diagnosed. Our objectives were to estimate the prevalence of late-stage breast cancer (TNM stage III or IV) at diagnosis overall, across age groups, and by ethnoracial and social strata (ie, self-reported ethnoracial group, as white, black, brown, Asian, or Indigenous, and educational level, marital status, and region of residence) across the study period, and compare these estimates with international data from high-income countries (Norway and the USA). We used logistic regression to estimate odds ratios (ORs) for late-stage versus early-stage (TNM stage I or II) breast cancer at diagnosis in relation to relevant exposures, either minimally adjusted (for age, year of diagnosis, and region of residence) or fully adjusted (for all patient, tumour, and health-care provider variables).

**Findings:**

We identified 247 719 women who were diagnosed with invasive breast cancer between Jan 1, 2001, and Dec 31, 2014, with a mean age at diagnosis of 55·4 years (SD 13·3), of whom 36·2% (n=89 550) identified as white, 29·8% (n=73 826) as black or brown, and 0·7% (n=1639) as Asian or Indigenous. Prevalence of late-stage breast cancer at diagnosis remained high throughout 2001–14, at approximately 40%, was inversely associated with educational level (p value for linear trend <0·0001), and was higher for women who identified as black (minimally adjusted OR 1·61, 95% CI 1·53–1·70; fully adjusted OR 1·45, 95% CI 1·38–1·54) and brown (minimally adjusted OR 1·26, 95% CI 1·22–1·30; fully adjusted OR 1·18, 1·14–1·23) than those who identified as white. The predicted prevalence of late-stage cancer at diagnosis was highest for women who were black or brown with little or no formal education (48·8%, 95% CI 48·2–49·5) and lowest for women who were white with university education (29·4%, 28·2–30·6), but both these prevalences were higher than that of all women diagnosed with breast cancer in Norway before the introduction of mammography screening (ie, 16·3%, 95% CI 15·4%–17·2% in 1970–74). Similar ethnoracial and social patterns emerged in analyses restricted to the age group targeted by screening (50–69 years).

**Interpretation:**

The persistently high prevalence of late-stage breast cancer at diagnosis across all ethnoracial and social strata in Brazil, although more substantially among the most disadvantaged populations, implies that early detection policies might have had little effect on breast cancer mortality so far, and highlights the need to focus primarily on timely diagnosis of symptomatic breast cancer rather than on screening for asymptomatic disease.

**Funding:**

Newton Fund, Research Councils UK, and Conselho Nacional das Fundações Estaduais de Amparo à Pesquisa.

## Introduction

Incidence of breast cancer in Brazil, an upper-middle-income country, has been increasing in the past decade, as in most other low-income and middle-income countries, reflecting population ageing and adoption of reproductive (eg, delayed age at first birth and fewer children than in previous generations) and lifestyle (eg, that lead to excess weight at postmenopausal ages) behaviours associated with an increased risk of developing the disease. Although incidence of the disease is still considerably lower in these countries than in most high-income countries (age-adjusted incidence: 59·5 per 100 000 women in Brazil *vs* 92·9 per 100 000 in the USA and 73·1 per 100 000 in Norway),^1^ mortality due to the disease is as high in Brazil as in many high-income countries (14·3 per 100 000 women in Brazil *vs* 14·9 per 100 000 in the USA and 12·5 per 100 000 in Norway),^1^ with mortality having increased across all age groups since 1979, contrasting substantially with decreases seen in most high-income countries since the early 1990s.^2^

**Research in context**

**Evidence before this study**

Incidence of breast cancer in Brazil has been increasing due to population ageing and adoption of risky lifestyles, and is expected to almost double by 2035. Early detection policies, with a focus on mammographic screening, but also including promotion of breast cancer awareness and annual clinical breast examinations for women aged 40 years and older, were introduced in 2004 by the Brazilian Government, but whether these policies are associated with a shift towards early-stage disease at diagnosis is unknown. We searched PubMed using no language or date restrictions on July 9, 2018, using the terms (breast cancer* OR breast neoplasm* OR breast carcinoma* OR breast sarcoma* OR breast tumor* OR breast tumour* OR breast malignanc*) AND (stage* OR clinical feature*) AND Brazil. We identified 298 publications and screened their titles and abstracts, and if relevant also their full text. A few papers had previously examined stage of breast cancer at diagnosis in Brazil, but none had specifically focused on assessing whether the 2004 control policies were associated with a decrease in the prevalence of late-stage disease at diagnosis across the various ethnoracial and social strata in Brazil.

**Added value of this study**

This study showed that the prevalence of late-stage breast cancer at diagnosis, at all ages and in the target screening age group (age 50–69 years), remained high at approximately 40% throughout 2001–14. Persistent ethnoracial and social differences were observed, with black and brown women with little or no formal education having the highest prevalence of late-stage disease at diagnosis and white women who were university educated having the lowest prevalence. However, white women who were university educated still had a substantially higher prevalence than all women diagnosed with breast cancer in Norway in the early 1970s—well before the introduction of mammographic screening in Norway. We estimated that an effective screening programme, with 80% coverage, could have prevented approximately 2500 deaths in Brazil in 2012, much less than the approximately 7500 deaths that could have been potentially prevented in the same year if 80% of stage III and IV cancers diagnosed in the previous 5 years had instead been stage II cancers.

**Implications of all the available evidence**

Mammographic screening programmes have been established in many high-income countries since the late 1980s, when findings from the earliest randomised trials were published. Since then, national and international advocacy groups have been exerting pressure on health systems in low-income and middle-income countries to emulate those in high-income countries by promoting mammographic screening. Our finding of a persistently high proportion of late-stage breast cancer at diagnosis in Brazil implies that early detection policies with a focus on mammographic screening might have had, so far, little effect on mortality from the disease and shows that what works in high-income countries does not necessarily work in low-income and middle-income countries. Shifting the focus of control policies from their current emphasis on mammographic screening towards the implementation of resource-appropriate approaches for early diagnosis of symptomatic disease could potentially prevent a much larger number of deaths from the disease than screening for asymptomatic disease.

Breast cancer is a potentially curable disease if diagnosed at an early stage. Advanced stage at diagnosis is difficult and costly to treat, and is associated with increased morbidity and poor survival in high-income countries^3^, ^4^ and in Brazil.^5^ Thus, a potential reason for the disproportionately high breast cancer mortality in Brazil is diagnosis at a late-stage.

A shift towards diagnosing breast cancer at an early stage is a necessary, but not sufficient, prerequisite for decreasing mortality from the disease. This shift can be achieved through downstaging (also known as stage migration—ie, by ensuring that symptomatic women are diagnosed and treated at an early stage) or through screening to detect asymptomatic disease. These two approaches are interconnected because the capability of a country's health system to appropriately manage symptomatic disease is a prerequisite to the introduction of an effective screening programme, since a health system that struggles to cope with symptomatic disease will be unable to deal with the additional burden of screen-detected suspicious lesions and subsequently confirmed asymptomatic cases. Nevertheless, early detection policies in Brazil, as in many low-income and middle-income countries, have tried to emulate those being currently implemented in high-income countries by focusing mainly on screening, with the Brazilian Ministry of Health recommending biennial mammographic screening for women aged 50–69 years since April, 2004.^6^ Promotion of breast cancer awareness and annual clinical breast examinations for women aged 40 years and older were also recommended but received less attention.

Ethnoracial and social disparities in early detection of and survival from breast cancer have been documented in many countries.^3^, ^7^, ^8^, ^9^, ^10^ Despite decreases in economic disparity in the past decade, Brazil still ranks among the countries with the highest levels of income inequality,^11^ with its population comprising several ethnoracial groups. Although studies have examined the prevalence of late-stage breast cancer at diagnosis and its correlates,^5^, ^12^, ^13^ none to our knowledge have focused on patterns across ethnoracial and social strata.

We aimed to use data from a nationwide network of hospital-based cancer registries in Brazil, covering 14 years from 2001 to 2014, to investigate whether the policies of early breast cancer detection introduced in 2004^6^ have led to a decrease in the prevalence of late-stage disease at diagnosis across the ethnoracial and social strata. Specifically, we aimed to identify patient-level, tumour-specific, and health system-related predictors of late-stage breast cancer at diagnosis; examine patterns in the prevalence of late-stage disease at diagnosis in Brazil overall and by ethnoracial, social, and age group, and compare these estimates with similar international data; and consider the implications of the findings for early detection policies.

## Methods

### Study design and participants

In this case-only study, women who had been diagnosed with breast cancer in Brazil were identified using a nationwide network of hospital-based cancer registries. The Brazilian unified health system (Sistema Único de Saúde [SUS]) was established by the government in 1988 to provide universal free access to health care. A network of SUS-affiliated hospital-based cancer registries (Registros hospitalares de cancer [RHC]) was set up in the early 1990s, and an electronic platform for standardised collection of data on each patient's sociodemographic characteristics, tumour features, and health-care access was adopted in 2000, the data from which are collected in the sisRHC database.^14^ The RHC network comprises two different sources: the Integrator Module of RHCs coordinated by the Brazilian National Cancer Institute, and the RHC of São Paulo state coordinated by Fundação Oncocentro of São Paulo (FOSP). These two sources use similar procedures except that the RHC of São Paulo state does not collect data on self-reported ethnoracial group, marital status, main basis for diagnosis, and centre where patient was first seen. Together these two sources comprise health-care providers with oncological accreditation located in each of the 26 Brazilian states and the Federal District.

The number of health-care providers and the proportion that submitted data to RHC varies from year to year. RHC comprised approximately 80% of all SUS-affiliated oncology accredited health-care providers in Brazil during the study period, although some health-care providers submitted data only intermittently for administrative reasons. However, geographical variations existed, with RHC covering the highest proportion of health-care providers in the southern region (eg, coverage in 2014 was 75% [52 of 69 providers] for the south, 70% [seven of ten] for the north, 68% [101 of 148] for the southeast, 62% [36 of 58] for the northeast, and 50% [13 of 26] for the central-west region; Azevedo e Silva G and Nogueira MC, unpublished).

Using anonymised electronic individual-level records from the sisRHC database, we identified all women diagnosed with an invasive breast cancer (ICD-10: C50;^15^ data on in-situ tumours were not available), aged 18–89 years in an SUS-affiliated health-care provider during 2001–14. Women with missing information on year of diagnosis or on age at diagnosis, and those diagnosed at a heath-care provider not affiliated with SUS or with unknown affiliation were excluded.

The study was approved by the ethics committee of the Instituto de Medicina Social, Universidade Estadual do Rio de Janeiro, Brazil, and the ethics committee of the London School of Hygiene & Tropical Medicine, London, UK. No permissions were needed to access and use data from these databases. The study protocol is provided in the appendix.

### Data extraction

We extracted individual patient-level, tumour-related, and health-care provider data for all eligible women. Patient-level data included their ethnoracial group as defined by the Brazilian Census (white, black, brown, Asian, or Indigenous); educational level (defined as less than primary education: ≤4 years of education; primary education: 5–9 years of education; secondary education: 8–12 years of education; and university education: >12 years of education); marital status; region of residence (each region is formed of smaller Unidades Federativas, and the Unidade Federativa of residence of a woman coincided with the Unidade Federativa where her health-care provider was located for all women); migration status (whether a woman had migrated out of her region of birth); year of diagnosis; and age at diagnosis. Tumour-related data included the stage of breast cancer (defined using the TNM Classification of Malignant Tumours [TNM]^16^) at diagnosis, basis for diagnosis, histological type of disease, and the presence of multiple tumours in the breast or breasts. Health-care provider data included type of health-care provider, level of SUS oncological accreditation (classified as Unidades de Assistência de Alta Complexidade em Oncologia [UNACON]**:** health-care providers with appropriate oncological resources to treat the five most common cancers in the country, including breast cancer; Centros de Alta Complexidade em Oncologia [CACON]: health-care providers with the necessary multidisciplinary resources to treat and manage any type of cancer; and other: health-care providers accredited to provide only specific oncological services—eg, surgical oncology or radiotherapy), type of management (ie, municipal or state), and type of service where the patient was first seen.

### Outcomes

Our objectives were to estimate the prevalence of late-stage breast cancer at diagnosis overall, across age groups, and by ethnoracial and social strata in Brazil from 2001 to 2014; to assess the extent to which prevalence of late-stage disease at diagnosis was affected by the early detection control policies introduced in Brazil in 2004; and to compare the prevalence of late-stage disease at diagnosis in Brazil with long-term data from high-income countries from before the introduction of mammographic screening to the most recent post-screening year for which such data were available.

Further objectives included estimation of the number of deaths that could potentially have been prevented in 2012 by mammographic screening under different scenarios including different levels of coverage and true effectiveness, and by downstaging if different proportions of women diagnosed with late-stage disease (TNM stage III or IV) in the previous 5 years had instead been diagnosed with TMN stage II disease.

### Statistical analysis

The distribution of TNM stage at diagnosis was examined overall and by variables related to patient, tumour, and health-care provider. We used logistic regression models to estimate odds ratios (ORs), with 95% CIs, for late stage (TNM stage III–IV) versus early stage (TNM stage I–II) breast cancer in relation to patient-related variables, first examined separately while controlling for year of diagnosis, age, and region of residence at diagnosis (minimally adjusted ORs), and then after further adjustment for all other variables (fully adjusted ORs). We did analyses overall and separately for the two main ethnoracial groups—ie, blacks and browns combined and whites. The number of Asian and Indigenous women was too small for meaningful stratum-specific analyses. Predicted probabilities (ie, predicted prevalence) of late-stage disease at diagnosis by educational level for women who identified as white and as black and brown were derived from the fully adjusted models fitted after averaging over the distribution of all of the explanatory variables other than education and ethnoracial group; we calculated SEs for these average predicted prevalences using the delta method.

We did all analyses for women of all ages and also restricted to the age group targeted by screening. To assess the robustness of the overall and stratum-specific results, we repeated our analyses on: multiple imputed data to address potential biases due to outcome and covariate data incompleteness; women who lived in the south; and women first diagnosed and treated in the health-care provider that submitted the data to RHC (a more stringent definition of incidence). We generated multiple imputation datasets using a fully conditional specification approach under the assumption of missingness at random.^17^ The imputation model included the outcome (stage at diagnosis), the main predictors of missingness (age, year of diagnosis, and region of residence), and all the variables contributing to the analysis models. Together these variables were also the main drivers of the variables affected by missingness. Missingness was assessed by comparing the distribution of all the variables between women with and without complete information. Additionally, to increase flexibility, the imputation model included interactions between year of diagnosis and all other variables. To control the Monte Carlo error of estimates and SEs, we generated 50 imputation sets. Patients from São Paulo state, whose registry did not record information on ethnoracial group, were excluded from the multiple imputation analyses to avoid extrapolation beyond the observed associations.

To compare the observed time trends in the prevalence of late-stage disease in Brazil with those from high-income countries, before and after the introduction of population-based mammographic screening, published data were extracted from the nationwide cancer registry of Norway, one of the few countries where such data have been collected.^18^, ^19^ Population-based mammographic screening was introduced gradually in Norway between 1995 and 2004. The US surveillance, epidemiology, and end results program (SEER) does not use the TNM classification;^16^ however, US tumour stage data, recoded according to the TNM classification, separately by age and by race, aggregated over the years 1988–2001, have been published. The published data are single estimates derived by aggregating data for the years 1988–2001 separately for women who identified as white and black, those aged 50–69 years, and all races combined.^4^ Opportunistic mammographic screening was introduced in the USA in the early 1980s. The reported prevalence over time of late-stage disease for these two countries were plotted together with the prevalence in our data, overall and in strata defined by ethnoracial group, educational level, and age.

We estimated the number of deaths that could have been prevented by an effective screening programme for women aged 50–69 years in 2012 (the year for which mortality data were available in Globocan^1^) in which mortality had been decreased by 20% due to mammographic screening, as observed in randomised controlled trials,^20^ while assuming different levels of coverage of screening (eg, 70%, 80%, 90%) and different true effectiveness in 2012. We also estimated the number of deaths that could have been potentially prevented in Brazil in 2012 by downstaging breast cancer among patients diagnosed in the previous 5 years, assuming that as low as 50% and as high as 80% of patients with late-stage disease had been diagnosed at TMN stage II instead, which reflects predominantly clinically detectable disease. Full details of calculations for number of deaths potentially prevented are in the appendix.

### Role of the funding source

The funders had no role in study design, data collection, data analysis, data interpretation, or writing of the report. The corresponding author had full access to all the data in the study and had final responsibility for the decision to submit for publication.

## Results

**Figure 1:**
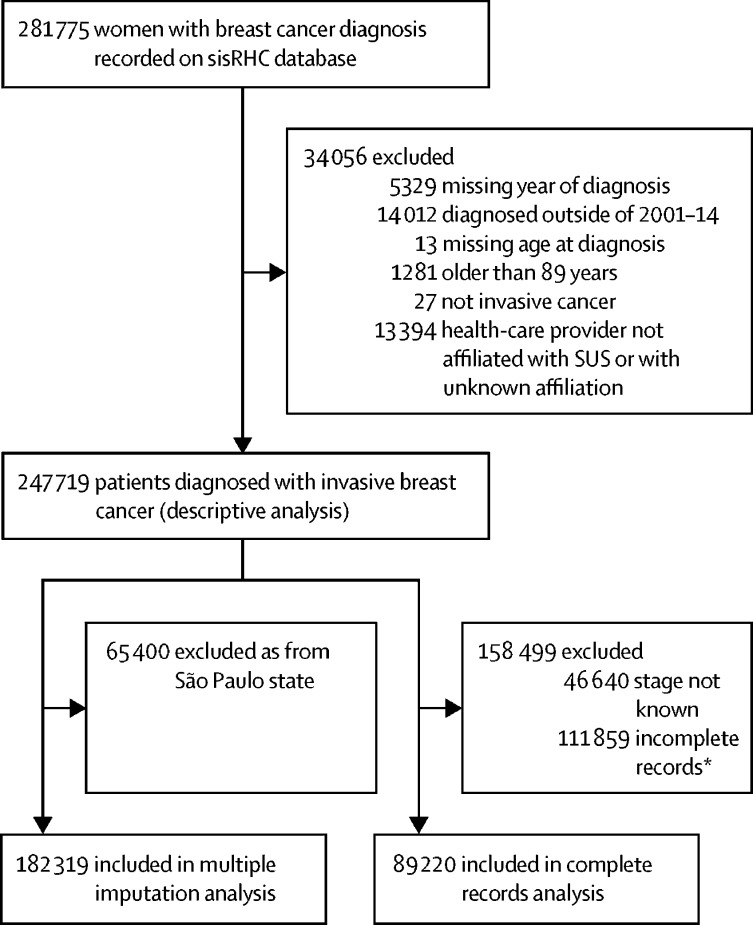
Participant selection *Missing data for one or more patient-level, tumour-level, or health-care provider-level variable.

Between Jan 1, 2001, and Dec 31, 2014, 247 719 women aged 18–89 years were diagnosed with an invasive breast cancer in SUS-affiliated health-care providers that contributed to RHC during this period (figure 1). Mean age at diagnosis was 55·4 years (SD 13·3). Of 247 719 women, 36·2% (n=89 550) were white and 29·8% (n=73 826) were black or brown, 35·1% (n=86 920) had no or less than primary formal education, and 30·3% (n=74 9870) were single, widowed, or divorced at the time of diagnosis. Most participants were first seen in a breast clinic (40·0%; n=99 024) or oncology service (19·0%; n=47 071) and were diagnosed with an invasive ductal carcinoma (81·6%; n=202 167). Other individual-level demographic, tumour-specific, and health-care provider variables are shown in table 1.

**Table 1:** Characteristics of the participants, overall and by stage at diagnosis

		**Early-stage disease (TNM stage I or II; n=120 322)**	**Late-stage disease (TNM stage III or IV; n=80 757)**	**All stages (including not known; n=247 719)**
**Patient characteristics**				
Ethnoracial group*				
	White	42 966 (63·1%)	25 115 (36·9%)	89 550 (36·2%)
	Black	4474 (49·8%)	4519 (50·3%)	11 083 (4·5%)
	Brown (Parda)	25 897 (54·4%)	21 689 (45·6%)	62 743 (25·3%)
	Asian	639 (54·1%)	542 (45·9%)	1498 (0·6%)
	Indigenous	63 (59·4%)	43 (40·6%)	141 (0·1%)
	Data missing	46 283 (61·6%)	28 849 (38·4%)	82 704 (33·4%)
Educational level†				
	None	6216 (49·6%)	6329 (50·5%)	15 828 (6·4%)
	Less than primary	34 157 (57·4%)	25 396 (42·6%)	71 092 (28·7%)
	Primary	17 990 (60·6%)	11 699 (39·4%)	35 512 (14·3%)
	Secondary	19 192 (62·4%)	11 555 (35·6%)	37 001 (14·9%)
	University	10 611 (69·5%)	4657 (30·5%)	18 743 (7·6%)
	Data missing	32 156 (60·4%)	21 121 (39·6%)	69 543 (28·1%)
Marital status				
	Married or living as married	38 768 (60·5%)	25 296 (39·5%)	83 783 (33·8%)
	Single, widowed, or divorced	31 974 (56·0%)	25 139 (44·0%)	74 987 (30·3%)
	Data missing	49 580 (62·1%)	30 322 (38·0%)	88 949 (35·9%)
Migrated out of region of birth				
	Yes	12 358 (56·5%)	9513 (43·5%)	25 902 (10·5%)
	No	100 438 (59·9%)	67 252 (40·1%)	208 184 (84·0%)
	Data missing	7526 (65·3%)	3992 (34·7%)	13 633 (5·5%)
Year of diagnosis				
	2001	4986 (59·1%)	3458 (41·0%)	9425 (3·8%)
	2002	6280 (61·5%)	3925 (38·5%)	11 382 (4·6%)
	2003	6296 (61·3%)	3971 (38·7%)	11 736 (4·7%)
	2004	6816 (60·9%)	4384 (39·1%)	12 891 (5·2%)
	2005	7381 (60·5%)	4827 (39·5%)	14 663 (5·9%)
	2006	7771 (59·5%)	5301 (40·6%)	15 976 (6·5%)
	2007	8816 (60·0%)	5876 (40·0%)	18 257 (7·4%)
	2008	9447 (59·0%)	6573 (41·0%)	20 314 (8·2%)
	2009	9963 (58·9%)	6962 (41·1%)	21 522 (8·7%)
	2010	10 039 (57·9%)	7316 (42·2%)	21 122 (8·9%)
	2011	10 693 (58·6%)	7551 (41·4%)	23 328 (9·4%)
	2012	10 959 (59·7%)	7400 (40·3%)	23 326 (9·4%)
	2013	11 451 (60·8%)	7395 (39·2%)	23 577 (9·5%)
	2014	9424 (61·8%)	5818 (38·2%)	19 200 (7·8%)
Age at diagnosis, years				
	18–39	11 365 (50·9%)	10 978 (49·1%)	27 407 (11·1%)
	40–49	30 187 (59·0%)	20 982 (38·9%)	62 910 (25·4%)
	50–59	32 957 (61·1%)	20 982 (38·9%)	66 295 (26·8%)
	60–69	25 772 (63·4%)	14 905 (36·6%)	50 072 (20·2%)
	70–89	20 041 (60·8%)	12 909 (39·2%)	41 035 (16·6%)
Region of residence at diagnosis				
	North	2895 (51·4%)	2743 (48·7%)	8001 (3·2%)
	Northeast	24 195 (55·5%)	19 371 (44·5%)	58 410 (23·6%)
	Central west	2256 (52·5%)	2043 (47·5%)	8542 (3·5%)
	Southeast	65 702 (60·5%)	42 902 (39·5%)	120 516 (48·7%)
	South	25 274 (64·9%)	13 698 (35·2%)	52 250 (21·1%)
**Tumour characteristics**				
Main basis for diagnosis‡				
	Histology	76 721 (58·8%)	53 728 (41·2%)	172 478 (69·6%)
	Other microscopic procedures	2246 (62·6%)	1341 (37·4%)	4918 (2·0%)
	Clinical examination	1614 (54·7%)	1337 (45·3%)	3947 (1·6%)
	Data missing	39 741 (62·0%)	24 351 (38·0%)	66 376 (26·8%)
Histological type§				
	Invasive ductal carcinoma	98 408 (59·5%)	67 025 (40·5%)	202 167 (81·6%)
	Invasive lobular carcinoma	6898 (61·0%)	4416 (39·0%)	13 491 (5·5%)
	Other	15 005 (61·8%)	9291 (38·2%)	31 946 (12·9%)
	Data missing	11 (30·6%)	25 (69·4%)	115 (0·1%)
Presence of multiple tumours in breast or breasts				
	Yes or possible	2997 (60·3%)	1972 (39·7%)	6492 (2·6%)
	No	74 180 (58·6%)	52 519 (41·5%)	168 180 (67·9%)
	Data missing	43 145 (62·2%)	26 266 (37·8%)	73 047 (29·5%)
**Health-care provider related variables**				
Type of health-care provider				
	General hospital	60 993 (61·6%)	37 958 (38·4%)	128 164 (51·7%)
	Specialised hospital	57 196 (58·3%)	40 986 (41·7%)	115 458 (46·6%)
	Other¶	2133 (54·1%)	1813 (46·0%)	4097 (1·7%)
Level of SUS oncological accreditation				
	CACON	54 219 (59·2%)	37 374 (40·8%)	108 184 (43·7%)
	UNACON	63 860 (60·3%)	42 128 (39·8%)	134 981 (54·5%)
	Other	496 (61·8%)	307 (38·2%)	1531 (0·6%)
	Not known	1747 (64·8%)	948 (35·2%)	3023 (1·2%)
Type of management				
	Municipal	60 997 (60·4%)	40 075 (39·7%)	129 986 (52·5%)
	State	37 406 (58·6%)	26 454 (41·4%)	72 589 (29·3%)
	Both	21 919 (60·6%)	14 228 (39·4%)	45 144 (18·2%)
Service where patient was first seen				
	Breast clinic	46 437 (59·4%)	31 735 (40·6%)	99 024 (40·0%)
	Oncology	19 029 (54·5%)	15 919 (45·6%)	47 071 (19·0%)
	Radiotherapy	6812 (66·9%)	3373 (33·1%)	13 473 (5·4%)
	Other‖	3960 (57·5%)	2925 (42·5%)	12 088 (4·9%)
	Data missing	44 084 (62·2%)	26 805 (37·8%)	76 063 (30·7%)

Data are n (%), with proportions in the early stage and late stage disease columns using the total number with that characteristic as the denominator, and in the total column using the total population as the denominator. SUS=Sistema Único de Saúde (Brazilian national public health service). CACON=Centros de Alta Complexidade em Oncologia. UNACON=Unidades de Assistência de Alta Complexidade em Oncologia.

* Based on self-reported ethnoracial group classified according to the Brazilian Census.

† Number of years of formal education: less than primary education ≤4 years; primary education 5–9 years; secondary education 8–12 years; university >12 years.

‡ Most important basis for breast cancer diagnosis.

§ Histological type classified according to the International Classification of Diseases for Oncology.^14^

¶ Includes stand-alone specialised clinics.

‖ Includes, among others, general surgery, gynaecology, and mammographic screening units.

**Figure 2:**
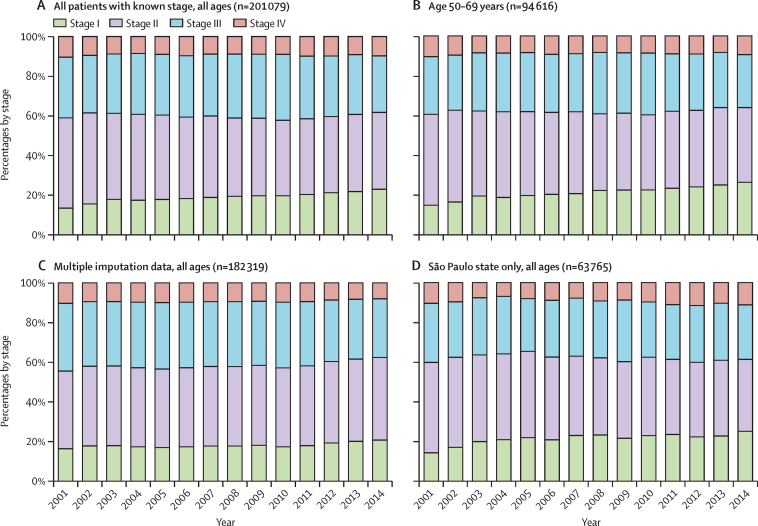
Breast cancer stage at diagnosis for all patients with known stage of all ages (A), those aged 50–69 years (B), from multiple imputation analysis (C), and in São Paulo of all ages (D), Brazil, 2001–14 Data from São Paulo state are not included in the multiple imputation analysis.

Stage at diagnosis was missing for 18·8% (46 640 of 247 719) of women diagnosed with invasive breast cancer. Of 201 079 with a known stage at diagnosis, 39 160 (19·5%) had stage I, 81 162 (40·4%) had stage II, 62 118 (30·9%) had stage III, and 18 639 (9·3%) had stage IV breast cancer. The proportion of women diagnosed with stage I disease increased slightly over time, but this increase was offset by a similar decrease in the proportion with stage II disease (figure 2A). Consequently, the proportion of women diagnosed with late-stage (III and IV) disease remained relatively constant (approximately 40% of people diagnosed per year; table 1) during the study period, except for a small decrease in 2011–14 (table 1; figure 2A). Similar patterns were observed in analyses restricted to women aged 50–69 years, multiple imputation data (all ages) that excluded data from São Paulo, and data from São Paulo only (figure 2B–D). Information on the extent of the primary tumour (the T in TNM classification) was missing for 69 535 (28·1%) women, but, among those with this information, tumour size at diagnosis was 2 cm or smaller for 26·8% (47 698 of 178 184), larger than 2 cm for 53·9% (95 982; including >5 cm for 15·5% [27 648]), and of any size but with extension to the skin or chest wall for 19·4% (34 504). Among women aged 50–69 years, 29·6% (25 102 of 84 027) had a tumour of 2 cm or smaller, 51·5% (43 282) had a tumour larger than 2 cm (including 13·4% [11 290] with a tumour >5 cm), and 18·6% (15 643) had a tumour of any size that extended to the skin or chest wall.

Because of missing covariate data, and notably because of a lack of collection of data on ethnoracial group, and marital status by health-care providers in São Paulo state, complete records were only available for 89 220 (36·0%) of 247 719 women (figure 1). The proportion of women with incomplete records (ie, data missing for at least one variable) varied according to age, year of diagnosis, and region at diagnosis (appendix). However, we saw little evidence of variation in the size of the associations between variables that were not affected by missingness (ie, age, year of diagnosis, region of diagnosis) and late-stage cancer at diagnosis, when estimated using all records with known stage of disease in subsets with complete and incomplete records for the other variables, and among the subset with incomplete records between those diagnosed and not diagnosed in São Paulo state (appendix).

Analyses into the association of patient ethnoracial and social characteristics with late-stage cancer at diagnosis yielded similar findings when minimally adjusted and fully adjusted models were fitted using data from women with complete records, indicating that mutual adjustment did not have a substantial effect on the estimates (table 2). The odds of late-stage cancer at diagnosis was higher for women who identified as black (minimally adjusted OR 1·61, 95% CI 1·53–1·70; fully adjusted OR 1·45, 1·38–1·54), brown (minimally adjusted OR 1·26, 95% CI 1·22–1·30; fully adjusted OR 1·18, 1·14–1·23), and Asian (minimally adjusted 1·29, 95% CI 1·13–1·48; fully adjusted OR 1·18, 1·03–1·36) than for those who identified as white, but not for women who identified as indigenous (although the estimates for this group had wide 95% CIs due to small numbers; table 2). Late-stage cancer at diagnosis was inversely associated with educational level (p value for linear trend <0·0001), with women who attended university having a fully adjusted OR of 0·41 (95% CI 0·38–0·44) compared with those who had no formal education. Single, widowed, or divorced women also had a higher odds of late-stage disease at diagnosis than women who were married or living as married (fully adjusted OR 1·23, 95% CI 1·19–1·26), as did women who had emigrated out of their region of birth than those who did not (fully adjusted OR 1·09, 1·03–1·15; table 2).

**Table 2:** Odds ratios for late-stage breast cancer in Brazil, 2001–14, overall and for the two main ethnoracial groups, from complete records and multiple imputation analyses, by patient ethnoracial and social characteristics

		Complete records analyses*					**Multiple imputation analyses**				
		All (minimally adjusted; n=89 220)†	All (full adjusted; n=89 220)†‡	Whites (full adjusted; n=43 860)‡	Blacks and browns (full adjusted; n=44 409)‡	p_interaction_	All (minimally adjusted; n=182 319)†	All (fully adjusted; n=182 319)	Whites (fully adjusted; n=89 550) ‡	Blacks and browns (fully adjusted; n=73 826)‡	p_interaction_
Ethnoracial group‡		..	..	..	..	NA	..	..	..	..	NA
	White	1 (ref)	1 (ref)	NA	NA	..	1 (ref)	1 (ref)	NA	NA	..
	Black	1·61 (1·53–1·70)	1·45 (1·38–1·54)	NA	NA	..	1·42 (1·36–1·48)	1·35 (1·30–1·42)	NA	NA	..
	Brown (Parda)	1·26 (1·22–1·30)	1·18 (1·14–1·23)	NA	NA	..	1·18 (1·15–1·21)	1·15 (1·11–1·18)	NA	NA	..
	Asian	1·29 (1·13–1·48)	1·18 (1·03–1·36)	NA	NA	..	1·17 (1·05–1·31)	1·14 (1·02–1·27)	NA	NA	..
	Indigenous	1·01 (0·63–1·64)	0·93 (0·57–1·50)	NA	NA	..	0·99 (0·69–1·44)	0·97 (0·66–1·41)	NA	NA	..
	p_heterogenity_	<0·0001	<0·0001	..	..	..	<0·0001	<0·0001	..	..	..
Educational level		..	..	..	..	0·31	..	..	..	..	0·38
	None	1 (ref)	1 (ref)	1 (ref)	1 (ref)	..	1 (ref)	1 (ref)	1 (ref)	1 (ref)	..
	Less than primary	0·71 (0·68–0·75)	0·73 (0·70–0·77)	0·73 (0·68–0·80)	0·73 (0·69–0·78)	..	0·80 (0·77–0·84)	0·81 (0·78–0·85)	0·83 (0·77–0·89)	0·79 (0·74–0·84)	..
	Primary	0·64 (0·61–0·68)	0·66 (0·63–0·70)	0·69 (0·63–0·76)	0·63 (0·59–0·68)	..	0·73 (0·70–0·77)	0·75 (0·71–0·79)	0·76 (0·71–0·82)	0·71 (0·66–0·76)	..
	Secondary	0·51 (0·48–0·54)	0·53 (0·51–0·57)	0·54 (0·50–0·60)	0·53 (0·49–0·57)	..	0·62 (0·59–0·66)	0·64 (0·61–0·68)	0·65 (0·60–0·70)	0·61 (0·57–0·65)	..
	University	0·39 (0·36–0·41)	0·41 (0·38–0·44)	0·43 (0·39–0·47)	0·38 (0·35–0·42)	..	0·51 (0·48–0·54)	0·52 (0·50–0·56)	0·53 (0·49–0·58)	0·48 (0·44–0·53)	..
	p_linear trend_	<0·0001	<0·0001	<0·0001	<0·0001	..	<0·0001	<0·0001	<0·0001	<0·0001	..
Marital status		..	..	..	..	0·080	..	..	..	..	0·44
	Married or living as married	1 (ref)	1 (ref)	1 (ref)	1 (ref)	..	1 (ref)	1 (ref)	1 (ref)	1 (ref)	..
	Single, widowed, or divorced	1·22 (1·19–1·26)	1·23 (1·19–1·26)	1·26 (1·21–1·31)	1·22 (1·18–1·27)	..	1·14 (1·12–1·17)	1·14 (1·12–1·17)	1·15 (1·11–1·19)	1·15 (1·11–1·19)	..
	p_heterogenity_	<0·0001	<0·0001	<0·0001	<0·0001	..	<0·0001	<0·0001	<0·0001	<0·0001	..
Migrated out of region of birth		..	..	..	..	0·13	..	..	..	..	0·41
	No	1 (ref)	1 (ref)	1 (ref)	1 (ref)	..	1 (ref)	1 (ref)	1 (ref)	1 (ref)	..
	Yes	1·09 (1·04–1·15)	1·09 (1·03–1·15)	1·08 (1·00–1·16)	1·09 (1·01–1·17)	..	1·08 (1·03–1·12)	1·07 (1·03–1·12)	1·05 (0·99–1·11)	1·07 (1·00–1·14)	..
	p_heterogenity_	0·0007	0·0012	0·044	0·026	..	0·0005	0·0009	0·12	0·035	..

Data are minimally or fully adjusted odds ratios with 95% CIs in parentheses, unless otherwise stated. Fully adjusted data are adjusted for all patient, tumour, and health-care provider variables listed in table 1. These analyses exclude data from São Paulo state. NA=not applicable. p_interaction_=p value for interaction with ethnoracial group.

* Complete records are those with non-missing data on breast cancer stage at diagnosis and all the variables listed in table 1.

† Minimally adjusted for age, year, and region of residence at breast cancer diagnosis.

‡ Self-reported ethnoracial group classified according to the Brazilian Census.

Similar findings were yielded when these complete-records analyses were restricted to patients diagnosed at age 50–69 years (appendix), those living in the south, and those who were first diagnosed and treated in the same health-care provider that submitted the data to RHC (appendix). Similar results were also obtained when analyses accounted for missing data via multiple imputation (table 2). The inverse trend in the odds of diagnosis with late-stage disease with increasing educational level, and the positive associations with being single, widowed, or divorced, and having emigrated out of the region of birth, were similar among people who identified as white and those who identified as black and brown, for both complete record and multiple imputation analyses (table 2).

The odds of late-stage cancer at diagnosis, after adjustment for year of diagnosis and age (minimal adjustment), was highest in the north and lowest in the south of the country, both overall and when analysed separately for people who identified as white and black and brown combined, and for complete records and multiple imputation analyses (table 3). There was evidence of a significant difference in the magnitude of the ORs for region of residence at diagnosis between the two ethnoracial groups (p_interaction_<0·0001 in the complete records analysis; p_interaction_=0·0030 for multiple imputation analysis), with women who identified as black and brown having greater odds of late-stage cancer at diagnosis than those who identified as white in the north of the country (table 3). As expected, the odds of late-stage cancer at diagnosis was higher among younger women, for whom the disease is most aggressive, than among older women, even after adjustment for region and year of diagnosis (table 3). We saw a pattern towards women who identified as black or brown having a decreased odds of late-stage cancer at diagnosis with later year of diagnosis compared with women who identified as white (p_interaction_<0·0001 for complete records analysis; p_interaction_=0·0007 for multiple imputation analysis). However, this interaction was driven by differences in the last and first year (when the analyses were restricted to 2002–13 p_interaction_=0·16 for complete records analysis and p_interaction_=0·35 for multiple imputation analysis; table 3).

**Table 3:** Odds ratios for late-stage breast cancer in Brazil, 2001–14, overall and for the two main ethnoracial groups, from complete records and multiple imputation analyses, by demographic characteristics

		Complete records analyses*					**Multiple imputation analyses**				
		All (minimally adjusted; n=89 220)†	All (fully adjusted; n=89 220)	Whites (fully adjusted; n=43 860)‡	Blacks and browns (fully adjusted; n=44 409)‡	p_interaction_	All (minimally adjusted; n=182 319)†	All (fully adjusted; n=182 319)	Whites (fully adjusted; n=89 550)‡	Blacks and browns (fully adjusted; n=73 826)‡	p_interaction_
Region of residence at diagnosis		..	..	..	..	<0·0001	..	..	..	..	0·0030
	North	1·95 (1·81–2·09)	2·00 (1·85–2·17)	1·85 (1·56–2·18)	2·06 (1·79–2·36)	..	1·69 (1·60–1·79)	1·68 (1·58–1·79)	1·75 (1·52–2·02)	1·76 (1·59–1·96)	..
	Northeast	1·41 (1·36–1·46)	1·25 (1·19–1·31)	1·35 (1·26–1·45)	1·26 (1·12–1·41)	..	1·48 (1·44–1·52)	1·32 (1·27–1·37)	1·38 (1·31–1·45)	1·25 (1·15–1·36)	..
	Central-west	1·79 (1·63–1·96)	1·38 (1·25–1·53)	1·26 (1·09–1·45)	1·54 (1·30–1·83)	..	1·65 (1·55–1·76)	1·31 (1·22–1·40)	1·38 (1·25–1·53)	1·21 (1·07–1·36)	..
	Southeast	1·28 (1·23–1·32)	1·17 (1·12–1·22)	1·19 (1·13–1·26)	1·19 (1·06–1·34)	..	1·33 (1·29–1·36)	1·22 (1·19–1·26)	1·22 (1·17–1·27)	1·19 (1·09–1·30)	..
	South	1 (ref)	1 (ref)	1 (ref)	1 (ref)	..	1 (ref)	1 (ref)	1 (ref)	1 (ref)	..
	p_heterogenity_	<0·0001	<0·0001	<0·0001	<0·0001	..	<0·0001	<0·0001	<0·0001	<0·0001	..
Age at diagnosis, years		..	..	..	..	0·22	..	..	..	..	0·021
	18–39	1·52 (1·45–1·59)	1·65 (1·57–1·73)	1·62 (1·51–1·74)	1·68 (1·57–1·79)	..	1·50 (1·45–1·56)	1·59 (1·53–1·65)	1·57 (1·48–1·67)	1·62 (1·53–1·71)	..
	40–49	1·10 (1·06–1·14)	1·15 (1·11–1·20)	1·11 (1·05–1·17)	1·18 (1·12–1·25)	..	1·09 (1·06–1·12)	1·13 (1·09–1·16)	1·09 (1·05–1·14)	1·17 (1·12–1·22)	..
	50–59	1 (ref)	1 (ref)	1 (ref)	1 (ref)	..	1 (ref)	1 (ref)	1 (ref)	1 (ref)	..
	60–69	0·91 (0·87–0·94)	0·84 (0·81–0·88)	0·85 (0·80–0·90)	0·84 (0·79–0·89)	..	0·91 (0·89–0·94)	0·87 (0·84–0·90)	0·87 (0·83–0·91)	0·86 (0·81–0·90)	..
	70–89	0·98 (0·94–1·02)	0·84 (0·80–0·88)	0·85 (0·80–0·91)	0·81 (0·76–0·87)	..	1·00 (0·97–1·04)	0·90 (0·87–0·93)	0·92 (0·88–0·97)	0·86 (0·81–0·91)	..
	p_heterogenity_	<0·0001	<0·0001	<0·0001	<0·0001	..	<0·0001	<0·0001	<0·0001	<0·0001	..
Year of diagnosis		..	..	..	..	0·0007§	..	..	..	..	0·0007§
	2001	0·96 (0·88–1·04)	0·90 (0·83–0·98)	0·82 (0·72–0·93)	1·00 (0·89–1·13)	..	0·95 (0·88–1·01)	0·92 (0·86–0·99)	0·86 (0·78–0·94)	1·01 (0·91–1·12)	..
	2002	0·97 (0·89–1·05)	0·94 (0·87–1·02)	0·97 (0·86–1·09)	0·93 (0·83–1·05)	..	0·88 (0·83–0·94)	0·89 (0·83–0·95)	0·87 (0·80–0·95)	0·93 (0·84–1·03)	..
	2003	1·02 (0·95–1·11)	0·99 (0·91–1·07)	0·93 (0·83–1·05)	1·08 (0·96–1·20)	..	0·92 (0·86–0·98)	0·91 (0·86–0·97)	0·90 (0·82–0·99)	1·01 (0·91–1·11)	..
	2004	1·07 (0·99–1·15)	1·04 (0·96–1·12)	0·98 (0·88–1·10)	1·08 (0·97–1·20)	..	0·95 (0·90–1·01)	0·95 (0·89–1·01)	0·93 (0·85–1·01)	1·01 (0·92–1·12)	..
	2005	1·06 (0·98–1·14)	1·04 (0·96–1·12)	1·02 (0·91–1·14)	1·03 (0·93–1·15)	..	1·01 (0·95–1·08)	1·01 (0·95–1·08)	1·03 (0·94–1·13)	1·04 (0·95–1·14)	..
	2006	1·06 (0·99–1·14)	1·05 (0·98–1·13)	1·09 (0·98–1·21)	1·02 (0·92–1·13)	..	1·01 (0·96–1·07)	1·01 (0·96–1·07)	1·04 (0·96–1·13)	1·00 (0·92–1·09)	..
	2007	1·01 (0·94–1·08)	0·99 (0·93–1·07)	0·98 (0·89–1·08)	1·01 (0·91–1·11)	..	0·99 (0·94–1·04)	0·98 (0·93–1·04)	1·00 (0·93–1·08)	0·99 (0·91–1·08)	..
	2008	1·05 (0·98–1·12)	1·04 (0·97–1·12)	1·06 (0·96–1·17)	1·02 (0·93–1·13)	..	1·03 (0·98–1·08)	1·02 (0·97–1·08)	1·05 (0·97–1·13)	1·00 (0·92–1·08)	..
	2009	1 (ref)	1 (ref)	1 (ref)	1 (ref)	..	1 (ref)	1 (ref)	1 (ref)	1 (ref)	..
	2010	1·08 (1·02–1·16)	1·10 (1·03–1·17)	1·09 (0·99–1·20)	1·10 (1·01–1·21)	..	1·10 (1·04–1·16)	1·10 (1·04–1·16)	1·11 (1·02–1·19)	1·12 (1·03–1·21)	..
	2011	1·02 (0·96–1·09)	1·04 (0·97–1·11)	1·02 (0·93–1·12)	1·03 (0·94–1·13)	..	1·03 (0·98–1·09)	1·04 (0·98–1·09)	1·03 (0·96–1·11)	1·04 (0·97–1·13)	..
	2012	0·93 (0·88–0·99)	0·95 (0·89–1·01)	0·97 (0·88–1·06)	0·92 (0·85–1·01)	..	0·95 (0·91–1·00)	0·96 (0·92–1·02)	0·97 (0·90–1·05)	0·94 (0·87–1·02)	..
	2013	0·90 (0·85–0·96)	0·92 (0·86–0·98)	0·91 (0·83–1·00)	0·91 (0·84–1·00)	..	0·91 (0·86–0·95)	0·92 (0·87–0·97)	0·91 (0·85–0·98)	0·91 (0·84–0·98)	..
	2014	0·85 (0·79–0·90)	0·88 (0·83–0·94)	0·96 (0·87–1·05)	0·81 (0·74–0·89)	..	0·87 (0·82–0·91)	0·89 (0·84–0·94)	0·96 (0·89–1·04)	0·83 (0·77–0·90)	..
	p_heterogeneity_	<0·0001	<0·0001	0·0001	<0·0001	..	<0·0001	<0·0001	<0·0001	<0·0001	..

Data are minimally or fully adjusted odds ratios with 95% CIs in parentheses, unless otherwise stated. Fully adjusted data are adjusted for all patient, tumour, and health-care provider variables listed in table 1. These analyses exclude data from São Paulo state. NA=not applicable. p_interaction_=p value for interaction with ethnoracial group.

* Complete records are those with non-missing data on breast cancer stage at diagnosis and all the variables listed in table 1.

† Minimally adjusted for age, year and region of residence at breast cancer diagnosis.

‡ Self-reported ethnoracial group classified according to the Brazilian Census.

§ This interaction was due to the values in the earliest and latest years (ie, 2001 and 2014); when analyses were restricted to 2002–13 p_interaction_=0·16 for complete-records and p_interaction_=0·35 for multiple imputation data.

**Figure 3:**
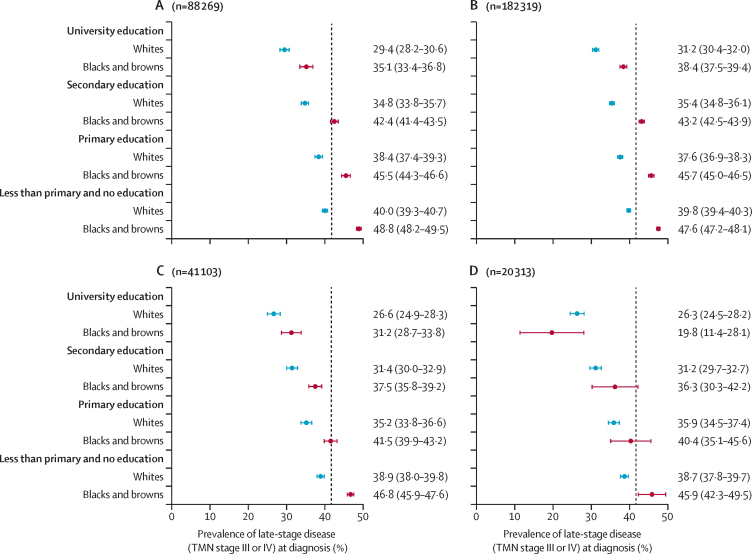
Estimated average prevalence of late-stage breast cancer at diagnosis in white women and black and brown women combined, by educational level (A) All regions and all ages, (C) all regions for those aged 50–69 years, and (D) in south Brazil for all ages as yielded by complete records analyses; and (B) for all regions and all ages by multiple imputation analyses. Data are average prevalence, with 95% CI in parentheses. Data from São Paulo state are not included in any of these analyses. Vertical dashed lines in panels A, B, and C indicate the observed average prevalence of late-stage disease at diagnosis in the complete records subset (n=88 269; 41·7%), and in panel B this line indicates the average prevalence of late-stage cancer at diagnosis from multiple imputed data (n=183 319; 41·3%).

Figure 3A shows the predicted prevalence of late-stage cancer at diagnosis for each education level, separately for women who identified as whites and those who identified as black and brown combined. In our analysis of women with complete records, across all ages and regions, we found an almost 20% absolute difference between the group with the highest predicted prevalence of late-stage cancer at diagnosis—black and brown women with no or less than primary education (48·8%, 95% CI 48·2–49·5)—and the group with the lowest predicted probability—white women with a university education (29·4%, 28·2–30·6; figure 3A). We saw a similar pattern in multiple imputation analysis of all ages in all regions, and in analyses restricted to those aged 50–69 years and those in the south of the country (figure 3B–D).

**Figure 4:**
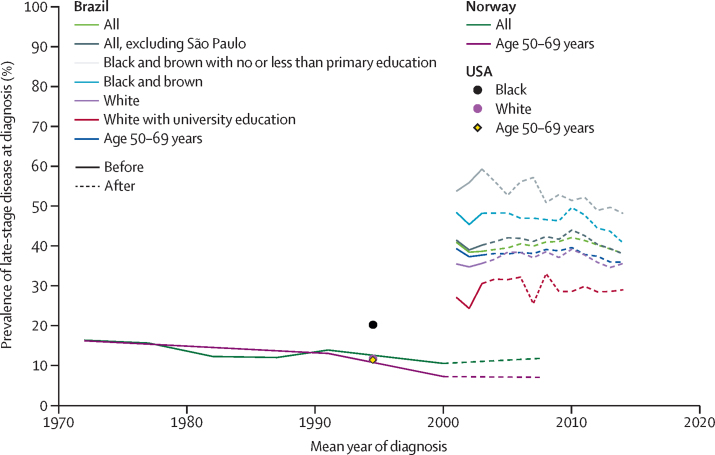
Prevalence of late-stage breast cancer at diagnosis in Brazil, 2001–14; in Norway, 1970–2010; and in the USA, 1988–2001, before and after introduction of mammographic screening Data for Brazil are for the whole country and selected population groups from 2001–14. Mammographic screening is population based in Norway and opportunistic in Brazil and the USA. For Norway, year of diagnosis in the published data was categorised as 1970–74, 1975–79, 1980–84, 1985–89, 1987–95, 1996–2004, and 2005–10; hence, estimates were plotted at the midpoint of each interval (eg, at 1972 for 1970–74 and at 2007·5 for 2005–10). Similarly, the estimates for the USA are aggregates for the whole time period. The US point estimates are for a time period after the introduction of opportunistic mammographic screening. Age specific data on stage of breast cancer at diagnosis by race were not provided in the US surveillance, epidemiology, and end results report.^4^

The overall prevalence of late-stage cancer at diagnosis remained high, at approximately 40%, in Brazil throughout 2001–14, although we observed a slight decrease since 2011 (figure 4). Nevertheless, the overall prevalence of late-stage disease at diagnosis in Brazil was more than twice that in Norway (16·3% [1043 of 6385; 95% CI 15·4–17·2] of women diagnosed with invasive breast cancer in 1970–74), well before the introduction of mammographic screening in Norway (figure 4). Even among the most privileged women in Brazil—ie, university educated white women—prevalence of late-stage cancer at diagnosis in 2014 was still considerably higher than among all women diagnosed with breast cancer in Norway in the early 1970s and among white and black women diagnosed in the USA in 1988–2001 (ie, after introduction of opportunistic screening in the USA; presented at a single timepoints in the middle of this period). Similar differences in the prevalence of late-stage cancer at diagnosis between Brazil and high-income countries emerged when analyses were restricted to women aged 50–69 years (figure 4).

Mortality due to breast cancer has increased across all age groups since 1979 in Brazil, contrasting substantially with the decreases seen in most high-income countries, including in Norway and the USA (appendix). The number of breast cancer deaths that might have been prevented in Brazil in 2012 by mammographic screening, assuming 80% coverage, appropriate follow-up, and management of abnormalities suspected to be cancerous, is approximately 2500 (appendix). Results for different screening coverages and true effectiveness scenarios are shown in the appendix. The number of breast cancer deaths that might have been prevented in 2012 if as many as 80% of all women diagnosed at stage III and IV in the previous 5 years had instead been diagnosed with stage II disease is approximately 7500. If this proportion had been as low as 50% of all women diagnosed at stage III and IV, approximately 4700 deaths might have been prevented (appendix).

## Discussion

In this study we showed that approximately 40% of women with breast cancer in Brazil were diagnosed at an advanced stage in 2001–14, a stage at which treatment options are more restricted and less effective and, hence, survival is poorer than at at earlier stages. The proportion of women diagnosed with advanced disease, overall and at the ages targeted by screening, remained high throughout the study period, although with a slight decrease since 2011, despite the introduction of early detection policies in 2004.^6^ We also identified persistently notable ethnoracial and social disparities, with the prevalence of late-stage cancer diagnoses being highest in women who identified as black and brown who had little or no formal education and lowest in women who were university educated and identified as white. Nevertheless, the predicted prevalence of late-stage disease among university educated white women diagnosed with breast cancer was still considerably higher than among all women diagnosed with cancer in Norway in 1970–74, well before the introduction of population-based mammographic screening in this country, and among white and black women in the USA in 1988–2001. Consistent with studies in sub-Saharan Africa^21^ and in the USA,^22^ single, widowed, or divorced women were more likely to be diagnosed at a late-stage of disease independent of their ethnoracial group or educational level. Having emigrated from their region of birth was also found to be independently associated with increased risks of late-stage disease at diagnosis.

Our study benefited from a large number of women with breast cancer who were identified through a nationwide network of SUS-affiliated health-care providers in Brazil, which covered approximately 80% of all oncology accredited health-care providers in the public system. A limitation of our study is the lack of data collected on in-situ tumours. Although over 20 population-based cancer registries exist in Brazil, covering predominantly urban areas, they do not collect information on tumour stage. Additionally, for logistic reasons, some health-care providers contribute data to RHC only intermittently. However, analysis restricted to the south of Brazil, the region with the highest RHC coverage, yielded ethnoracial differences in the prevalence of late-stage breast cancer at diagnosis. Some women (eg, poor women living in remote areas) might have died from breast cancer without having ever contacted a health-care provider where a definitive diagnosis of the disease could be made. Such women could not be captured by the RHC network, but their exclusion from our analysis would have led, if anything, to an underestimation of the prevalence of diagnosis of late-stage disease. Nevertheless, the observed prevalence of late-stage disease at diagnosis is consistent with that reported by a Brazilian multicentre retrospective study of 3142 women with breast cancer.^5^ Self-reported ethnoracial group is prone to misclassification but a good overall agreement with genomic ancestry was found by a study in Brazil.^23^ Outcome and covariate data were missing for various subgroups of women, particularly from São Paulo state. Reassuringly, our findings were robust across the various subsets of women examined and in multiple imputation analyses, although the results from the multiple imputation analysis were conditional on the assumption that the variables included in the imputation step were sufficient to render missingness at random. If this assumption was untrue, selection bias would affect the results. However, the multiple imputation analyses included the most important drivers of missingness in this sisRHC administrative dataset (ie, age, year of diagnosis, and region of residence at diagnosis).

The persistently high prevalence of late-stage breast cancer at diagnosis at all ages and at ages targeted by screening indicates that early detection policies, including mammographic screening, are likely to have had, so far, little effect on mortality from this disease, and is consistent with the continuing rise in breast cancer mortality in Brazil.^2^ Late-stage breast cancer at diagnosis might be the result of an aggressive fast-growing tumour or the consequence of long delays between symptom onset and diagnosis. RHC does not collect data on markers of tumour aggressiveness but some studies^24^, ^25^ have shown that the receptor subtype distribution in Brazil is broadly similar to that found in high-income countries.^26^ The frequency of more aggressive molecular subtypes (eg, hormone-receptor-negative breast cancer) is higher in the USA among black women than white women,^8^, ^27^, ^28^ but no similar differences in the distribution of tumour subtypes between black and white women has been observed in Brazil.^24^, ^25^ Furthermore, the large educational gradient observed in both white women and black and brown women in our study is consistent with ethnoracial differences in late-stage cancer at diagnosis being mainly driven by social rather than biological factors. Ethnoracial disparities in health-care access,^29^, ^30^, ^31^ including access to mammographic screening,^32^ have been observed in Brazil to be driven by discrimination and lack of support policies for women who are black or brown.^33^, ^34^, ^35^

Delays from symptom recognition to diagnosis of 3 months or longer are associated with later stage at presentation and poorer survival than earlier diagnoses.^36^ In high-income countries, the time interval from symptom recognition to a diagnosis is usually less than 30 days,^37^, ^38^ but this interval appears to be much longer in Brazil (median of about 7–8 months).^9^ Tumour size distribution in our study is consistent with pronounced delays in diagnosis after a tumour is palpable and, hence, clinically symptomatic. Only 27% of women in our study had invasive tumours that were 2 cm or smaller and 15% were larger than 5 cm. By contrast, 65·9% of women with invasive tumours in the USA in 1988–2001 were 2·2 cm or smaller, and 5·8% were larger than 5·2 cm at diagnosis.^4^ A tumour growth model^39^ has predicted that, on average, a tumour of 2 cm (the average size when a tumour first becomes clinically palpable) takes 12 months to double in size, and 24 months to reach 6 cm in size. Although such models rely on several assumptions (eg, breast cancer subtype distribution and biology being similar to those in high-income countries),^39^ they indicate that the distribution of tumour size in Brazil is consistent with long delays in diagnosis after symptom onset.

In high-income countries, mammographic screening was introduced at a time when symptomatic disease had already been successfully downstaged, as illustrated by historical data from Norway where the prevalence of late-stage disease at diagnosis was only around 15% in 1970–74—ie, at a time when the health system was able to appropriately manage symptomatic disease and could thus cope with the additional burden of screen-detected asymptomatic cases. In Brazil, as in many other low-income and middle-income countries,^40^ early detection policies have focused mainly on mammographic screening^6^ despite weak health systems. Consequently implementation is opportunistic rather than population based, with coverage in the target age group for SUS-affiliated women (ranging from 27%, based on SUS data,^41^ to 51%, based on self-reports in a Brazilian National Health Survey for 2013^42^) well below the 70% minimum coverage for a programme to be effective,^43^ and poor follow-up of screened women with radiological findings suspected to be cancerous (27% for women aged 50–59 years and 63% for those aged 60–69 years in 2010 based on SUS data^41^), indicating poor effectiveness. At the same time, resources are being diverted to screen women outside the target age group, particularly those younger than 50 years.^41^ In high-income countries, the introduction of organised mammographic screening has led to a substantial increase in the proportion of small (in situ and stage I) tumours being diagnosed and a concomitant decrease in the proportion of large tumours.^44^, ^45^ Data on in-situ tumours were not available in our study, but the proportion of stage I tumours increased only modestly in the age group targeted for screening throughout the study period, in line with the programme having low coverage.

We estimate that about 7500 deaths due to breast cancer might have been prevented in Brazil in 2012 if 80% of women with breast cancer who had been diagnosed at stage III or IV in the previous 5 years had instead been diagnosed with stage II disease. By contrast, mammographic screening, assuming an 80% coverage and appropriate follow-up of radiologically suspicious abnormalities might have prevented approximately 2500 deaths in 2012. Although these estimates are based on many assumptions, and far from ideal estimates of breast cancer incidence, mortality, and survival (eg, no availability of national stage-specific survival estimates overall or by ethnoracial stratum), they highlight the potential benefit of investing in downstaging relative to screening. Notably, 53·0% of all women diagnosed with invasive breast cancer during the study period, corresponding to 55·6% of those diagnosed at stages III and IV, were diagnosed outside the screening target age group (ie, 50–69 years). Even in countries with a high-coverage population-based screening programme, most women with breast cancer present symptomatically (eg, in the UK in 2007, only 32% of all breast cancers, and 56% in the targeted age group of 50–69 years, were detected via screening).^46^

With the incidence of breast cancer in Brazil projected to almost double by 2035,^1^ timely diagnosis of symptomatic disease will be key to decreasing mortality due to the disease. Although the 2018 Brazilian Government guidelines^47^ put an emphasis on mammographic screening, they also highlight the need for early diagnosis of women who are symptomatic.^47^ The findings from our study indicate that priority should be given to women who are symptomatic by implementing resource-appropriate strategies to ensure timely access to diagnosis and treatment, particularly among those who are disadvantaged. Implementation of effective population-based screening programmes requires a strong health system that is able to appropriately manage symptomatic disease and the capability to provide access to high-quality mammography, high coverage of the target population, timely access for women with screen-detected abnormalities suspected to be cancerous to appropriate diagnostic and treatment services, and comprehensive built-in quality assurance mechanisms. The findings from this study show that even an upper-middle-income country such as Brazil, which provides universal free access to health care, struggles to achieve these requirements.

For the **Integrator Module** see https://irhc.inca.gov.br/RHCNet/

For the **RHC of São Paulo state** see www.fosp.saude.sp.gov.br

For the **ethnoracial composition of the Brazilian population** see https://sidra.ibge.gov.br/Tabela/3175

## Data sharing

All data are publicly available at https://irhc.inca.gov.br/RHCNet/.
