# Supplementary material for: Ethnoracial and social trends in breast cancer staging at diagnosis in Brazil, 2001–14: a case only analysis
Source: Lancet Glob Health. 2019 May 13;7(6):e784–97. doi: 10.1016/S2214-109X(19)30151-2 (PMC6527520; doi:10.1016/S2214-109X(19)30151-2)
Supplement: Supplementary appendix [file mmc1.pdf]

THE LANCET
Global Health

Supplementary appendix

This appendix formed part of the original submission and has been peer reviewed. We post it as supplied by the authors.

Supplement to: dos-Santos-Silva I, De Stavola B L, Renna Junior N L, et al. Ethnoracial and social trends in breast cancer staging at diagnosis in Brazil, 2001–14: a case only analysis. *Lancet Glob Health* 2019; **7:** e784–797.

Appendix

Table of contents

	**Page**
Text S1. Study protocol	2
Text S2. RECORD statement	5
Text S3: Number of Breast Cancer Deaths That Could Potentially Have Been Prevented by Mammographic Screening and by Downstaging of Symptomatic Disease in Brazil, 2012	9
Table S1. Minimally-Adjusted Odds Ratios for Late-Stage Breast Cancer by Variables Not Affected by Missingness Estimated in Different Patient Subsets, Brazil, 2001-2014	14
Table S2. Minimally- and Fully-Adjusted Odds Ratios for Late-Stage Breast Cancer by Patients' Ethnoracial and Social Characteristics, women aged 50-69 at diagnosis	15
Table S3. Fully-Adjusted Odds Ratios for Late-Stage Breast Cancer by Patients' Social Characteristics, Among Patients Resident in the South region, and Among Patients Who Fulfil a More Stringent Definition of "Incident" Cases; Brazil, 2001-2014, complete records only	16
Figure S1. Distribution of Age and Year of Diagnosis in Each Region by Record Completeness	17
Figure S2. Age-specific trends in breast cancer mortality in Brazil (1979-2013), Norway (1953-2013) and the USA (1950-2013)	18

Appendix S1_Study Protocol

Breast cancer stage at diagnosis in Brazil

Background

Breast cancer incidence in Brazil, as in most low/middle-income countries (LMICs), has been increasing in recent years, reflecting population aging as well as adoption of riskier reproductive and lifestyle behaviours. Albeit incidence is still considerably lower than in high income countries (HICS), mortality from the disease is as high as in many HICs (14·3/100,000 in Brazil vs. 14·9/100,000 in the US and 12·5/100,000 in Norway).[1] Breast cancer is a potentially curable disease if diagnosed at an early stage. Advanced stage at diagnosis is more difficult and costly to treat, and is associated with increased morbidity and poor survival in HICs, and Brazil. Thus, a potential reason for the disproportionately high breast cancer mortality in Brazil is late-stage at diagnosis.

A shift towards diagnosing breast cancer at an early stage is a necessary, albeit not sufficient, prerequisite for reducing mortality from the disease. This can be achieved through downstaging (also known as stage migration), i.e. by ensuring that symptomatic women are diagnosed and treated at an early stage, or through screening to detect asymptomatic disease. Early detection control policies in Brazil, as in many other LMICs, have tried to emulate those being currently implemented in HICs by focusing mainly on the latter with the Ministry of Health recommending, since 2004, biannual mammography for all women aged 50-69 years. However, it is unclear whether they have led to reductions in late-stage disease at diagnosis. Promotion of breast cancer awareness and yearly clinical breast examinations for women aged ≥40 years were also recommended but received less attention.

Study aims

The specific aims of the study are to:

- Estimate the prevalence of late-stage breast cancer at diagnosis, overall and across the various ethnoracial and social strata in the country during the period 2001-2014;
- Investigate the extent to which prevalence of late-stage disease was affected by the early detection control policies introduced in 2004;
- Compare the prevalence of late-stage in Brazil with long-term data from high-income countries stretching back to the period prior to the introduction of mammographic screening in these settings; and
- Consider the implications of the findings for cancer control policies in Brazil and other LMICs facing an increasing breast cancer burden.

Data sources

The Brazilian unified health system (Sistema Único de Saúde, SUS) was established by the government in 1988 to provide universal free access to health care. A network of SUS-affiliated hospital-based cancer registries (RHC) was set up in the 1990s, and an electronic platform for standardised collection of data on each patient's socio-demographic characteristics, tumour features and health care access (sisRHC), including the variables listed in Table 1, was adopted in 2000.[2] The RHC network comprises two different sources: the Integrator Module of RHCs (ttps://irhc.inca.gov.br/RHCNet/) coordinated by the Brazilian National Cancer Institute, and the RHC of São Paulo state (www.fosp.saude.sp.gov.br) coordinated by Fundação Oncocentro of São Paulo (FOSP). They use similar procedures except that the latter does not collect data on self-reported ethnicity/race, marital status, main basis for diagnosis, and centre where patient was first seen. Together they comprise health care providers (HCP) with oncological accreditation located in each of the 26 Brazilian states and the Federal District. The sisRHC data are publically available on ttps://irhc.inca.gov.br/RHCNet/. Although there are over 20 local population-based cancer registries in Brazil, covering mainly urban populations, none of them collects data on tumour stage at diagnosis.

Anonymised individual-level records will be extracted from the sisRHC database for all women with a breast cancer diagnosis. The study will comply with the RECORD statement.

To compare trends in the prevalence of late-stage breast cancer in Brazil, overall and in different ethnoracial and social strata, with those in high-income countries we will search for published long-term data, stretching back to the time period prior to the introduction of screening in these settings, from population-based cancer registries which have collected such historical data.

Study design

Case-only analysis of individual-level record data from the sisRHC database.

Study population

Women in the sisRHC database will be eligible if:

- they were diagnosed with an invasive breast cancer (ICD-10:C50[3]) in a SUS-affiliated HCP during 2001-2014;
- they were aged 18-89 years at the time of the diagnosis.

Women will be excluded if:

- year of breast cancer diagnosis is missing or outside the 2001-2014 range
- age at diagnosis is missing or outside the 18-89 range
- their diagnosis is a non-invasive breast cancer (TNM[4] behaviour codes: 3, 6, and 9)
- their breast cancer was diagnosed in a health care provider (HCP) not affiliated with SUS, or if the affiliation of the HCP at the time of the diagnosis was unknown.

The selection process, including the number of women excluded by reason, will be provided in a detailed flowchart.

Outcome and exposures

The outcome variable of interest would be stage of breast cancer at diagnosis as defined by the Classification of Malignant Tumours (TNM).[4]

The exposure variables will be classified as:

- *Patient-related variables*, i.e. ethnicity/race, educational level, marital status, migration out of region of birth, age at diagnosis, year at diagnosis, and region of residence at diagnosis.

- *Tumour-related* variables – main basis for diagnosis, histological type, and presence of multiple tumours in the breast(s)

- *HPC-related* – type of HCP, level SUS oncological accreditation, type of management, and type of service where that patient was first seen.

The patient-related variables will be the primary exposures of interest. Tumour-related and HCP-related variables will be taken as covariates in the analysis.

Statistical analysis

Comprehensive data checks will be performed to identify inconsistencies of the data, and data missingness patterns will be investigated.

The distribution of stage at diagnosis will be examined overall, and by patient-, tumour-, and HPC-related variables. Prevalence of late stage at diagnosis (TNM III and IV combined), overall and across ethnoracial and social strata, will be estimated and compared with similar international data.

Logistic regression models will be used to estimate odds ratios (ORs), with 95% confidence intervals (CIs), for late-stage (TNM III/IV) versus early stage (TNM I/II) breast cancer in relation to patient-related variables, before and after adjustment for covariates. Analyses will be conducted overall, and separately for each ethnoracial stratum.

To assess the robustness of the overall and stratum-specific results the analyses will be repeated on:

(i) *Multiple imputed data (MI)* to address potential biases due to outcome and covariate data incompleteness. MI data will be generated using a fully conditional specification approach under the assumption of missingness at random (MAR).[5] For flexibility, the imputation model will include the main predictors of missingness, the outcome (stage), and all the variables contributing to the analysis models, including interactions between year of diagnosis and all other variables. To control the Monte Carlo error of estimates and standard errors, 50 or more imputation sets will be generated.

(ii) *Women aged 50-69 years at diagnosis*, the age-group targeted by mammographic screening;

(iii) *Women resident in the South*, the region with the highest RHC coverage;

(iv) *Using a more stringent definition of "incidence"*, i.e. restricted to women first diagnosed and treated in the HCP that submitted the data to RHC, to ensure that the recorded stage was actually ascertained at diagnosis.

References

1. Ferlay J, Soerjomataram I, Dikshit R, Eser S, Mathers C, Rebelo M, et al. *GLOBOCAN 2012 v1.0, Cancer Incidence and Mortality Worldwide*, IARC CancerBase No. 10 [Internet]. International Agency for Research on Cancer. Lyon, France: 2013. Available from: http://globocan.iarc.fr, accessed on 20/12/2013.
2. Departamento de Ciência e Tecnologia. Secretaria de Ciência e Tecnologia e Insumos Estratégicos do Ministério da Saúde. Information integration of Brazilian cancer registries. *Rev Saúde Pública* 2007; **41**: 865–8.
3. World Health Organisation (WHO). *International Classification of Diseases, 10th Revison* (ICD-10). WHO: Geneva, Switzerland, 2010.
4. American Joint Committee on cancer (AJCC). *Breast Cancer Staging, 7th Edition*. American Cancer Society, 2009.
5. White IR, Royston P, Wood AM. Multiple imputation using chained equations: Issues and guidance for practice. *Stat Med* 2011; **20**; 30: 377-99.

Deviation from the original protocol

The only deviation from the above protocol was the conduct of additional analyses to estimate the number of breast cancer deaths that could have been prevented in Brazil, in 2012 (Text S3):

- if an efficient population-based mammographic screening programme was in place;

- if cases diagnosed at stage III and IV in the previous 5 years had been diagnosed at stage II instead (i.e. if symptomatic disease had been successfully downstaged).

Text S2. The RECORD statement

	Item No.	STROBE items	Location in manuscript where items are reported	RECORD items	Location in manuscript where items are reported
**Title and abstract**					
	1	(a) Indicate the study's design with a commonly used term in the title or the abstract (b) Provide in the abstract an informative and balanced summary of what was done and what was found		RECORD 1.1: The type of data used should be specified in the title or abstract. When possible, the name of the databases used should be included.<br><br>RECORD 1.2: If applicable, the geographic region and timeframe within which the study took place should be reported in the title or abstract.<br><br>RECORD 1.3: If linkage between databases was conducted for the study, this should be clearly stated in the title or abstract.	Abstract, Methods subsection<br><br>Abstract, Methods subsection<br><br>Not applicable
Background rationale	2	Explain the scientific background and rationale for the investigation being reported			Introduction, paragraphs 1-3
Objectives	3	State specific objectives, including any prespecified hypotheses			Introduction, last paragraph
Study Design	4	Present key elements of study design early in the paper			Methods section
Setting	5	Describe the setting, locations, and relevant dates, including periods of recruitment, exposure, follow-up, and data collection			Methods section, study design and participants subsection
Participants	6	*(a) Cohort study* - Give the eligibility criteria, and the sources and methods of selection of participants. Describe methods of follow-up<br><br>*Case-control study* - Give the eligibility criteria, and the sources and methods of case ascertainment and control selection. Give the rationale for the choice of cases and controls		RECORD 6.1: The methods of study population selection (such as codes or algorithms used to identify subjects) should be listed in detail. If this is not possible, an explanation should be provided.<br><br>RECORD 6.2: Any validation studies of the codes or algorithms used to select the population should be referenced. If validation was conducted for this study and	Methods section; Figure 1<br><br>Not applicable

		Cross-sectional study - Give the eligibility criteria, and the sources and methods of selection of participants<br><br>*(b) Cohort study* - For matched studies, give matching criteria and number of exposed and unexposed<br><br>*Case-control study* - For matched studies, give matching criteria and the number of controls per case		not published elsewhere, detailed methods and results should be provided.<br><br>RECORD 6.3: If the study involved linkage of databases, consider use of a flow diagram or other graphical display to demonstrate the data linkage process, including the number of individuals with linked data at each stage.	Not applicable
Variables	7	Clearly define all outcomes, exposures, predictors, potential confounders, and effect modifiers. Give diagnostic criteria, if applicable.		RECORD 7.1: A complete list of codes and algorithms used to classify exposures, outcomes, confounders, and effect modifiers should be provided. If these cannot be reported, an explanation should be provided.	Methods, statistical analysis subsection.
Data sources/ measurement	8	For each variable of interest, give sources of data and details of methods of assessment (measurement).<br><br>Describe comparability of assessment methods if there is more than one group			Methods, statistical analysis subsection; Tables 1-3
Bias	9	Describe any efforts to address potential sources of bias			Methods, statistical analysis subsection, paragraph 2
Study size	10	Explain how the study size was arrived at			Figure 1
Quantitative variables	11	Explain how quantitative variables were handled in the analyses. If applicable, describe which groupings were chosen, and why			Statistical analysis section; Tables 1-3
Statistical methods	12	(a) Describe all statistical methods, including those used to control for confounding<br><br>(b) Describe any methods used to examine subgroups and interactions<br><br>(c) Explain how missing data were addressed<br><br>(d) *Cohort study* - If applicable, explain how loss to follow-up was addressed<br><br>*Case-control study* - If applicable, explain how matching of cases and controls was addressed<br><br>*Cross-sectional study* - If applicable, describe analytical methods taking account of sampling strategy<br><br>(e) Describe any sensitivity analyses			Methods, statistical analysis subsection
Data access and cleaning methods		..		RECORD 12.1: Authors should describe the extent to which the investigators had access	Methods, study design and data extraction subsections

				to the database population used to create the study population.<br><br>RECORD 12.2: Authors should provide information on the data cleaning methods used in the study.	Appendix S1<br><br>Appendix S1
Linkage		..		RECORD 12.3: State whether the study included person-level, institutional-level, or other data linkage across two or more databases. The methods of linkage and methods of linkage quality evaluation should be provided.	Not applicable
**Results**					
Participants	13	(a) Report the numbers of individuals at each stage of the study (*e.g.*, numbers potentially eligible, examined for eligibility, confirmed eligible, included in the study, completing follow-up, and analysed)<br><br>(b) Give reasons for non-participation at each stage.<br><br>(c) Consider use of a flow diagram		RECORD 13.1: Describe in detail the selection of the persons included in the study (*i.e.,* study population selection) including filtering based on data quality, data availability and linkage. The selection of included persons can be described in the text and/or by means of the study flow diagram.	Results, Study design and participants subsection.<br><br>Figure 1
Descriptive data	14	(a) Give characteristics of study participants (*e.g.*, demographic, clinical, social) and information on exposures and potential confounders<br><br>(b) Indicate the number of participants with missing data for each variable of interest<br><br>(c) *Cohort study* - summarise follow-up time (*e.g.*, average and total amount)			Results section.<br><br>Table 1;<br><br>Figure S1
Outcome data	15	*Cohort study* - Report numbers of outcome events or summary measures over time<br><br>*Case-control study* - Report numbers in each exposure category, or summary measures of exposure<br><br>*Cross-sectional study* - Report numbers of outcome events or summary measures			Results section;<br><br>Table 1;<br><br>Figure 2
Main results	16	(a) Give unadjusted estimates and, if applicable, confounder-adjusted estimates and their precision (e.g., 95% confidence interval). Make clear which confounders were adjusted for and why they were included<br><br>(b) Report category boundaries when continuous variables were categorized			Tables 2 and 3;<br><br>Figures 3-4<br><br>Text S3

		(c) If relevant, consider translating estimates of relative risk into absolute risk for a meaningful time period			
Other analyses	17	Report other analyses done—e.g., analyses of subgroups and interactions, and sensitivity analyses			Figures 4<br><br>Tables S1-S3
**Discussion**					
Key results	18	Summarise key results with reference to study objectives			Discussion section, first paragraph
Limitations	19	Discuss limitations of the study, taking into account sources of potential bias or imprecision. Discuss both direction and magnitude of any potential bias		RECORD 19.1: Discuss the implications of using data that were not created or collected to answer the specific research question(s). Include discussion of misclassification bias, unmeasured confounding, missing data, and changing eligibility over time, as they pertain to the study being reported.	Discussion section.
Interpretation	20	Give a cautious overall interpretation of results considering objectives, limitations, multiplicity of analyses, results from similar studies, and other relevant evidence			Discussion section
Generalisability	21	Discuss the generalisability (external validity) of the study results			Discussion section.
**Other Information**					
Funding	22	Give the source of funding and the role of the funders for the present study and, if applicable, for the original study on which the present article is based			Methods section, Role of the funders subsection;<br><br>Acknowledgment section
Accessibility of protocol, raw data, and programming code		..		RECORD 22.1: Authors should provide information on how to access any supplemental information such as the study protocol, raw data, or programming code.	Original protocol given in Text S1. Access to the raw data given in the Methods section, study design and participants sub-section. Access to programing code available upon request to the authors.

Text S3: Number of Breast Cancer Deaths That Could Potentially Have Been Prevented by Mammographic Screening and by Downstaging of Symptomatic Disease in Brazil, 2012

I. Number of breast cancer deaths that could be prevented by mammographic screening

We aimed to estimate the number of breast cancer deaths that might have been prevented by mammographic screening in 2012, the more recent year for which data are available.

Data

Data on number of female breast cancer deaths in Brazil, by age, were extracted from GLOBOCAN for the year 2012:[1]

Age (yrs)	0-14	15-39	40-44	45-49	50-54	55-59	60-64	65-69	70-74	75+	All
No. deaths	0	1,296	1,114	1,626	1,972	2,033	1,836	1,666	1,611	3,258	16,412

We assumed that the protective effect of mammographic screening carries over to the ages beyond 69 years, i.e. beyond the upper limit of the age-group targeted by screening.

Calculations

- Using the data from the table above, the total number of deaths in Brazil, in 2012, among women aged 50+ was 12,376.
- We considered two extreme scenarios:
  A. Mammographic screening had actually led to a 20% reduction in mortality from breast cancer in 2012 among women aged 50+ who had been screened, with this percentage corresponding to the effect estimated in a meta-analysis of randomized controlled trials with 100% coverage.[2]
  B. Mammographic screening had actually led to no reduction in mortality from breast cancer in 2012 among these same women.

- Under scenario A and with 100% coverage, the total number of breast cancer deaths that might have been prevented by screening is (12,376 x 1/0.80 - 12,376) = 3,094.
- Under scenario A and with 70% coverage, the total number of breast cancer deaths that might have been prevented by screening is [12,376 x (0.70 x 1/0.8 + 0.30)] - 12,376 = 2,166
- Under scenario B and with 100% coverage, the total number of breast cancer deaths that would have been prevented by screening would be (12,376 x 0.20) = 2,475.
- Under scenario B and with 70% coverage, the total number of breast cancer deaths that would have been prevented by screening would be (12,376 x 0.70) x 0.20 = 1,732.

**Coverage of the screening programme:**	**No. of breast cancer deaths potentially prevented by screening in 2012:**	
	**Scenario A**	**Scenario B**
100%	3,094	2,475
90%	2,785	2,228
80%	2,475	1,980
70%	2,166	1,732

II. Number of breast cancer deaths that could be prevented by downstaging

We aimed to estimate the number of breast cancer deaths that would have been prevented in Brazil, in 2012, if varying percentages of women diagnosed with stage III/IV breast cancer in the previous 5 years had been diagnosed at stage II instead of stage III/IV. To

achieve this, we have used breast cancer mortality and incidence data, as well as estimated breast cancer survival rates and hazard ratios, which were obtained from different sources and then combined with data from our study.

Data

We used the following information:

1) No. female breast cancer deaths by age, Brazil, 2012, from GLOBOCAN[1] as shown in the table above

2) No. breast cancer incident cases (all ages), Brazil, 2012, from GLOBOCAN[1]
N=67,316

3) Stage-specific survival rates among breast cancer patients in São Paulo, as reported for 2000-2005 in FOSP, [4] Figure 4. From the figure we derived the stage III 1-year survival rates to be 92%.

4) Estimated age and stage mutually-adjusted mortality hazard ratios (HRs) among breast cancer patients in São Paulo, as reported for 2000-2005 in FOSP, [4] with stage 0 and age 20-29y as reference

Variable	Category	HR
Stage	0	1
	I	4.307
	IIA	13.147
	IIB	23.433
	III	61.804
	IV	193.849
Age (years)	20-29	1
	30-39	1.094
	40-49	0.808
	50-59	0.992
	60-69	1.001
	70-79	1.005
	80+	1.250

5) Observed frequency of women diagnosed with an invasive breast cancer at ages 18-89 years in SUS-affiliated hospital-based cancer registries (RHC) in Brazil during the years 2001-2014, by age group and stage (restricted to patients for whom stage was known)

Age at diagnosis (years)	Stage				
	I	II	III	IV	Total
18-39	2,536	8,829	8,615	2,363	22,343
40-49	8,977	21,210	16,620	4,363	51,170
50-59	11,360	21,597	16,231	4,751	53,939
60-69	9,449	16,323	11,247	3,658	40,677
70-89	6,838	13,203	9,405	3,504	32,950
Total	39,160	81,162	62,118	18,639	201,079

Calculations

These consisted of several steps:

(a) *Estimation of the distribution of female breast cancer incident cases in Brazil in 2012 by age and stage*

Assuming the average distribution by age and stage found in our data for the period 2001-2014 is representative of the patients diagnosed in 2012, we have used the observed distribution to create a table of age- and stage-frequencies for the

2012 incident cases (using weights defined by the ratio of the number of incidence cases in 2012 over the total number of cases in 2011-2014, that is: 67,316/201,079=0.335).

This reweighting procedure gives the following estimated distribution:

Age at diagnosis (years)	Stage				
	I	II	III	IV	Total
18-39	848.99	2,955.72	2,884.08	791.07	7,479.85
40-49	3,005.27	7,100.55	5,563.94	1,460.62	17,130.40
50-59	3,803.03	7,230.11	5,433.72	1,590.51	18,057.40
60-69	3,163.28	5,464.51	3,765.20	1,224.60	13,617.60
70-89	2,289.18	4,420.02	3,148.55	1,173.05	11,030.80
Total	13,109.70	27,170.90	20,795.50	6,239.85	67,316

(b) *Derivation of the age- and stage 1-year survival rates after breast cancer diagnosis in Brazil*

We used the reported 1-year survival rates for women diagnosed with stage III in 2012, $S_{III}(1)$ to derive the corresponding stage III mortality rate λ_{III}. Inverting the known relationship between survival rates and hazard rates, $S_{III}(1)=\exp(-\lambda_{III})$, we derived:

$$\boldsymbol{\lambda_{III}=-\log(S_{III}(1))=0.0834}$$

We also used the reported mutually adjusted age- and stage-specific HRs that were reported using stage 0 and age 20-29 as references, to derive the HRs where stage III and age 40-49 are the references (by taking the ratio of the each original HR over the HR for new reference) .

**Stage**	**HR**
I	0.070
II	0.295
III	1
IV	3.137

**Age**	**HR**
20-29	1.238
30-39	1.353
40-49	1
50-59	1.228
60-69	1.239
70-79<br>80+	1.395

Assuming that the hazard rate for stage III, λ_{III} derived above, applies to the most frequent age group (age 40-49), we calculated survival probabilities for all combinations of age and stage and for a selection of times, using the relationship between hazard rates and survival probabilities (also used above):

$$\boldsymbol{S_{stage,age}(t)=\exp(-\lambda_{stage,age}*t),}$$

where the derived values of $\boldsymbol{\lambda_{stage,age}=\lambda_{III}*HR_{stage}*HR_{age}}$, the HRs are given above, and t=1,2,3,4,5. Given the coarser classification of younger women in the GLOBOCAN data, we assumed the 30-39 year HRs also to be valid for all 18-39 year olds.

For example, the derived 1-year stage- and age-specific survival probabilities are:

Age at diagnosis (years)	Stage I	II	III	IV
18-39	0.992	0.967	0.893	0.702
40-49	0.994	0.976	0.920	0.770
50-59	0.993	0.970	0.903	0.725
60-69	0.993	0.970	0.902	0.723
70-89	0.992	0.966	0.890	0.694

Similar values were calculated for the 2-, 3-, 4- and 5-year survival probabilities.

(c) *Calculation of the number of lives saved under different scenarios, in comparison with the status quo.*

In this last step, we used the data above to predict the number of breast cancer deaths in 2012 and then consider alternative scenarios where some of the women who were stage III or IV at diagnosis are migrated to stage II.

We first predict the number of deaths in 2012 assuming:

(i) they originated from incidence cases recorded in the previous 5 years (i.e. from 2007 to 2011)
(ii) the number of incident cases in each year from 2007 and 2012 is the same as that observed in 2012
(iii) the age- and stage-distribution of these incident cases is the same as that observed in our data

These calculations allow us to assess whether the assumptions above, as well as the calculations in (a) and (b), are plausible. Below we reproduce the predicted numbers of deaths in 2012 arising from the 5 cohorts of incident cases whose diagnosis was in 2007, 2008, 2009, 2010, and 2011.

Predicted number of deaths in 2012 by stage and age among those diagnosed in 2011 (using 1-year survival probabilities)

Age at diagnosis (years)	Stage I	II	III	IV
18-39	6.7	97.1	307.9	235.9
40-49	17.4	173.1	445.1	336.1
50-59	27.0	215.8	528.7	436.8
60-69	22.7	164.5	369.5	338.9
70-89	18.5	149.6	345.8	358.7

Predicted number of deaths in 2012 by stage and age among those diagnosed in 2010 (using 2-year survival probabilities)

Age at diagnosis (years)	Stage I	II	III	IV
18-39	6.6	93.9	275.0	165.6
40-49	17.3	168.8	409.5	258.8
50-59	26.8	209.3	477.3	316.8
60-69	22.5	159.6	333.3	245.1
70-89	18.3	144.5	307.8	249.0

etc.

The total number of predicted deaths across the 5 cohort is 17,408.1, which is very close to the observed number of 16,412, considering the likely overestimation of incident cases.

We then calculated the number of breast cancer deaths in 2012 that could have been saved under different scenarios. The scenarios are:

Scenario 1: 100% of women with an initial stage III or IV at diagnosis were instead diagnosed as stage II.

Scenario 2: 80% of women with an initial stage III or IV at diagnosis were instead diagnosed as stage II.

Scenario 3: 50% of women with an initial stage III or IV at diagnosis were instead diagnosed as stage II.

In scenario 1, all women originally diagnosed at stage III/IV would have migrated to stage II. Hence, for these women the expected number of deaths was calculated using the survival rate of stage II women of the same age, instead of the survival rate of their given stage. For the other scenarios (1 to 3), the survival rate for stage II was reassigned only for, respectively, 80% and 50% of the original stage III/IV patients.

Scenario	Proportion of stage III/IV cancers down-staged:	Tot. No. predicted deaths in 2012	Change from "status quo"	
			Difference	%
0	"status quo"	17,408.1		
1	100%	7,982.4	9,425.7	54.1
2	80%	9,867.6	7,540.6	43.3
3	50%	12,695.3	4,712.9	27.1

References

1. Ferlay J, Soerjomataram I, Disshit R, Eser S, Mathers G, Rebelo M, et al. *GLOBOCAN 2012 v1.0, Cancer Incidence and Mortality Worldwide: IARC CancerBase No. 10 [Internet]*. International Agency for Research on Cancer. Lyon, France: 2013. Available from: http://globocan.iarc.fr (accessed on 20/12/2013).
2. Independent UK Panel on Breast Cancer Screening. The benefits and harms of breast cancer screening: an independent review. Lancet 2012; **380**: 1778-86.
3. World Health Organisation (WHO). *Cancer Control: Knowledge in to Action. WHO Guide to Effective Programmes. Module 3, Early Detection.* WHO, Geneva, Switzerland: 2007.
4. Fundação Oncocentro de São Paulo (FOSP), *Sobrevida de Pacientes com Câncer no Estado de São Paulo: Seis Anos de Seguimento pelo Registro Hospitalar de Câncer.* Cadernos FOSP, Volume 5. Secretary de Estado da Saúde & FOSP, São Paulo, 2009.

Table S1. Minimally-Adjusted Odds Ratios for Late-Stage Breast Cancer by Variables Not Affected by Missingness Estimated in Different Patient Subsets, Brazil, 2001-2014: All women with known stage; Subset with Complete Records; and Subset with Incomplete Data, Stratified According to Whether Women Were Non-Resident or Resident in São Paulo State

Variables with non-missing data	All women with known stage [a] (N=201,079)		Subset with complete records [b] (N=89,220)		Subset with incomplete records, [c] excluding São Paulo [d] (N=48,094)		Subset with incomplete records, [c] São Paulo state [d] (N=63,765)	
**Variable**	**OR [e]**	**95% CI**	**OR [e]**	**95% CI**	**OR [e]**	**95% CI**	**OR [e]**	**95% CI**
**Year of diagnosis**								
2001	**0·98**	0·93, 1·04	**0·96**	0·88, 1·04	**0·96**	0·86, 1·07	**1·01**	0·92, 1·11
2002	**0·90**	0·85, 0·94	**0·97**	0·89, 1·05	**0·79**	0·71, 0·87	**0·91**	0·84, 0·99
2003	**0·90**	0·86, 0·95	**1·02**	0·95, 1·11	**0·79**	0·71, 0·88	**0·86**	0·79, 0·94
2004	**0·92**	0·88, 0·97	**1·07**	0·99, 1·15	**0·82**	0·74, 0·91	**0·85**	0·78, 0·92
2005	**0·95**	0·90, 0·99	**1·06**	0·98, 1·14	**0·98**	0·90, 1·08	**0·80**	0·73, 0·87
2006	**0·99**	0·94, 1·03	**1·06**	0·99, 1·14	**0·96**	0·88, 1·05	**0·91**	0·83, 0·99
2007	**0·97**	0·92, 1·01	**1·01**	0·94, 1·08	**0·96**	0·88, 1·05	**0·89**	0·82, 0·97
2008	**1·00**	0·96, 1·05	**1·05**	0·98, 1·12	**1·00**	0·92, 1·08	**0·93**	0·85, 1·01
2009	**1**		**1**		**1**		**1**	
2010	**1·05**	1·00, 1·09	**1·08**	1·02, 1·16	**1·13**	1·04, 1·23	**0·91**	0·84, 0·99
2011	**1·02**	0·97, 1·06	**1·02**	0·96, 1·09	**1·09**	1·00, 1·19	**0·95**	0·88, 1·03
2012	**0·98**	0·94, 1·02	**0·93**	0·88, 0·99	**1·05**	0·96, 1·15	**1·02**	0·94, 1·10
2013	**0·93**	0·89, 0·97	**0·90**	0·85, 0·96	**0·96**	0·87, 1·05	**0·97**	0·90, 1·05
2014	**0·90**	0·86, 0·94	0·85	0·79, 0·90	0·96	0·87, 1·06	0·96	0·88, 1·04
P_t	*0·08*		*<0·001*		*<0·001*		*<0·001*	
**Age at diagnosis (years)**								
18-	**1·49**	1·45, 1·54	**1·52**	1·45, 1·59	**1·41**	1·32, 1·50	**1·52**	1·43, 1·61
40-	**1·08**	1·06, 1·11	**1·1**	1·06, 1·14	**1·04**	0·99, 1·09	**1·10**	1·05, 1·15
50-	**1**		**1**		**1**		**1**	
60-	**0·91**	0·89, 0·94	**0·91**	0·87, 0·94	**0·93**	0·88, 0·98	**0·92**	0·87, 0·96
70-	**1·02**	0·99, 1·05	**0·98**	0·94, 1·02	1·02	0·96, 1·08	**1·06**	1·01, 1·12
P_t	*<0·001*		*<0·001*		*<0·001*		*<0·001*	
**Region of residence at diagnosis [f]**								
North	**1·71**	1·61, 1·81	**1·95**	1·81, 2·09	**1·35**	1·23, 1·48	-	
North-East	**1·45**	1·41, 1·49	**1·41**	1·36, 1·46	**1·50**	1·43, 1·57	-	
Central-West	**1·64**	1·54, 1·75	**1·79**	1·63, 1·96	**1·48**	1·35, 1·61	-	
South-East	**1·21**	1·18, 1·24	**1·28**	1·23, 1·32	**1·40**	1·33, 1·46	-	
South	**1**		**1**		**1**		-	
P_{het}	*<0·001*		*<0·001*		*<0·001*			
**Type of HCP management**								
Municipal	**1**		**1**		**1**		**1**	
State	**1·07**	1·05, 1·09	**0·98**	0·95, 1·02	**1·09**	1·03, 1·16	**1·36**	1·31, 1·40
Both	**0·91**	0·88, 0·93	**0·86**	0·83, 0·89	**0·86**	0·82, 0·90	- [g]	
P_{het}	*<0·001*		*<0·001*		*<0·001*		*<0·001*	

CI: confidence interval; HCP: health care provider; OR: odds ratio; P_{het}: P for heterogeneity; P_t: P for linear trend

[a] This represents the whole eligible study population with known stage which, by design, had no-missing data on year, age and region of residence at diagnosis (see Figure S1). Data on type of HCP management was also available for all these women.

[b] Subset of eligible women with complete data on all the analysis covariates listed in Table 1: year of diagnosis, age at diagnosis, region of residence at diagnosis, race/ethnicity, educational level, marital status, whether they had migrated out of the region of birth, the main basis for diagnosis, histological type, presence of multiple tumour, type of HCP, level of SUS (Sistema Único de Saúde) oncological accreditation, type of HCP management, and service where the patient was first seen.

[c] Subset of eligible women with incomplete data on at least one of the variables listed in footnote b.

[d] Records from São Paulo state were, by design, incomplete as its RHC used a different form which did not include collection of data on the following variables: ethnicity, marital status, main basis for breast cancer diagnosis, and centre where patient was first seen.

Table S2. Minimally- and Fully-Adjusted Odds Ratios for Late-Stage Breast Cancer by Patients' Ethnoracial and Social Characteristics, women age 50-69 at diagnosis[a]: Overall and for the Two Main Ethnoracial Groups, Brazil, 2001-2014, complete records only [b]

Variables	All (N=41,510)		All (N=41,510)		Whites [c] (N=20,971)		Blacks/Browns [c] (N=20,132)	
	Minimally-adjusted		Fully-adjusted		Fully-adjusted		Fully-adjusted	
	**OR [d]**	**95% CI**	**OR [e]**	**95% CI**	**OR [e]**	**95% CI**	**OR [e]**	**95% CI**
Ethnoracial group [c]								
White	**1**		**1**		n/a		n/a	
Black	**1.63**	1.51, 1.77	**1.47**	1.36,1.59	n/a		n/a	
Brown ('Parda')	**1.25**	1.19, 1.31	**1.17**	1.11,1.23	n/a		n/a	
Asian	**1.18**	0.95, 1.45	**1.07**	0.87,1.33	n/a		n/a	
Indigenous	1.03	0.45, 2.40	**1.02**	0.43,2.39	n/a		n/a	
P_{het}	*<0.001*		*<0.001*					
Educational level [f]								
None	**1**		**1**		**1**		**1**	
Less than primary	**0·72**	0.67,0.77	**0.74**	0.69,0.79	**0.67**	0.60,0.76	**0.78**	0.72,0.85
Primary	**0·65**	0.58,0.68	**0.65**	0.60,0.70	**0.61**	0.53,0.69	**0.66**	0.59,0.73
Secondary	**0·51**	0.47,0.55	**0.53**	0.49,0.58	**0.49**	0.43,0.56	**0.56**	0.50,0.62
University	**0·39**	0.36,0.43	**0.41**	0.37,0.45	**0.39**	0.34,0.45	**0.41**	0.36,0.47
P_t	*<0.001*		*<0·001*		*<0.001*		*<0.001*	
Marital status								
Married/living as married	**1**		**1**		**1**		**1**	
Single/widowed/divorced	**1·22**	1.17,1.27	**1.22**	1.17,1.27	**1.27**	1.20,1.35	**1.20**	1.13,1.27
P_{het}	*<0.001*		*<0.001*		*<0.001*		*<0.001*	
Migrated out of region of birth [g]								
No	**1**		**1**		**1**		**1**	
Yes	**1·10**	1.02,1.18	**1.12**	1.04,1.20	**1.13**	1.01,1.25	**1.08**	0.96,1.20
P_{het}	*0.01*		*<0.01*		*0.03*		*0.19*	

CI: Confidence interval; OR: odds ratio; P_{het}: P for heterogeneity; P_t: P for linear trend

[a] The target age-group for mammographic screening.

[b] Records with complete data on stage at diagnosis and on all variables listed in Table 1. Consequently, records from São Paulo state were excluded (see Methods section).

[c] Based on self-reported race and skin colour classified according to the Brazilian Census (https://sidra.igge.gov.br/Tabela/3175).

[d] Adjusted for age, year and region of residence at breast cancer diagnosis.

[e] Adjusted for all patient-, tumour- and health care provider-related variables listed in Table 1.

[f] No. years of formal education – less than primary education: ≤ 4 years; primary education: 5-9 years; secondary education: 8-12 years; university: >12 years.

[g] Each region is formed by smaller Unidade Federativas (UF). The UF of residence of the patient was the same as the UF where her HCP was located for all participants.

Table S3. Fully-Adjusted Odds Ratios for Late-Stage Breast Cancer by Patients' Social Characteristics, Among Patients Resident in the South region, and Among Patients Who Fulfil a More Stringent Definition of "Incident" Cases; Brazil, 2001-2014, complete records only.[a]

**Variables**		**South region [b] (N=20,366)**		**"Incident" cases only [c] (N=40,318)**	
		**Fully-adjusted [d]**		**Fully-adjusted [d]**	
		**OR**	**95% CI**	**OR**	**95% CI**
Ethnoracial group [e]					
White		**1**		**1**	
Black		**1·21**	1·03, 1·44	**1·40**	1·30, 1·52
Brown ('Parda')		**1·10**	0·95, 1·28	**1·15**	1·09, 1·21
Asian		**1·27**	0·69, 2·32	**1·02**	0·84, 1·24
Indigenous		**1·51**	0·39, 5·84	**1·46**	0·70, 3·06
	P_{het}	*0·12*		*<0·001*	
Educational level [f]					
None		**1**		**1**	
Less than primary		**0·73**	0·64,0·84	**0·73**	0·68,0·78
Primary		**0·64**	0·55,0·73	**0·69**	0·64,0·75
Secondary		**0·49**	0·42,0·56	**0·55**	0·51,0·60
University		**0·38**	0·32,0·45	**0·43**	0·39,0·48
	P_t	*<0·001*		*<0·001*	
Marital status					
Married/living as married		**1**		**1**	
Single/widowed/divorced		**1·28**	1·20,1·36	**1·24**	1·19,1·29
	P_{het}			*<0·001*	
		<0·001			
Migrated out of region of birth [g]					
No		**1**		**1**	
Yes		**1·07**	0·94,1·21	**1·03**	0·95,1·11
	P_{het}	*0·32*		*0.51*	

CI: Confidence interval; OR: odds ratio; P_{het}: P for heterogeneity; P_t: P for linear trend

[a] Records with complete data on stage at diagnosis and on all variables listed in Table 1. Consequently, records from São Paulo state were excluded (see Methods section).

[b] The region with the highest RHC coverage (see Methods section).

[c] Subset of patients whose cancer was diagnosed for the first time in the HCP that submitted the data (a more stringent definition of incidence – see Methods section).

[d] Adjusted for all patient-, tumour- and health care provider-related variables listed in Table 1.

[e] Based on self-reported race/skin colour classified according to the Brazilian Census (https://sidra.igge.gov.br/Tabela/3175)

[f] No. years of formal education – less than primary education: ≤ 4 years; primary education: 5-9 years; secondary education: 8-12 years; university: >12 years.

[g] Each region is formed by smaller Unidade Federativas (UF). The UF of residence of the patient was the same as the UF where her HCP was located for all participants.

Figure S1. Distribution of Age and Year of Diagnosis in Each Region by Record Completeness

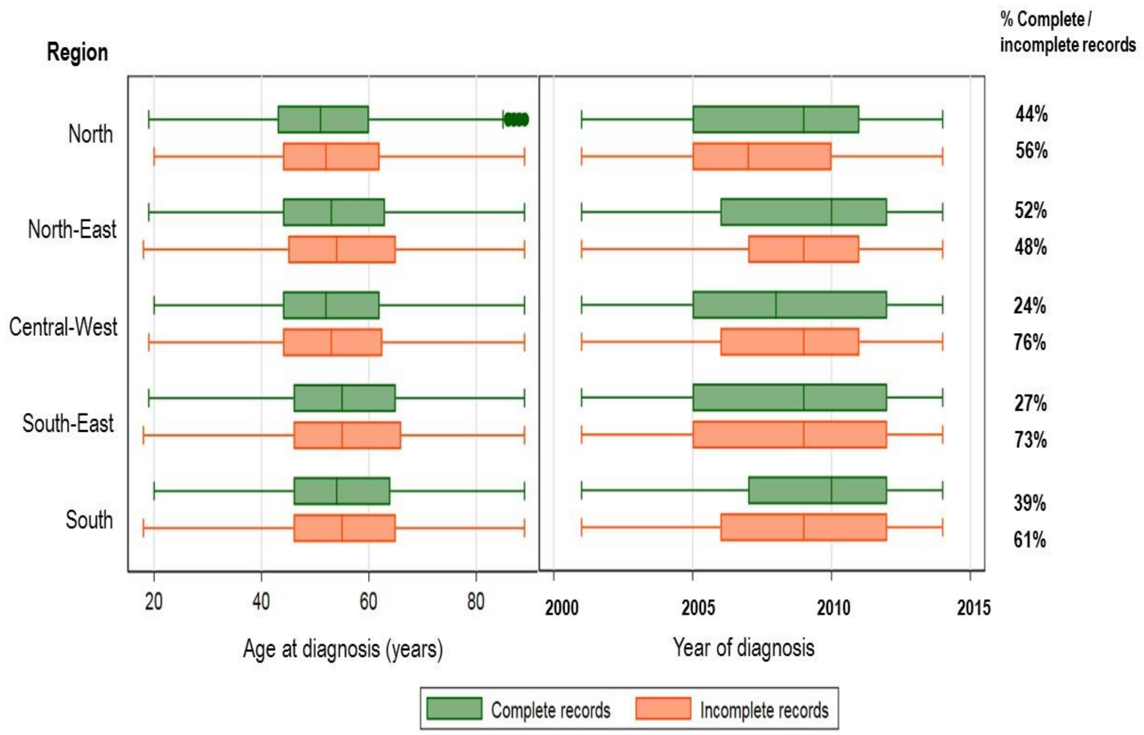

Complete records are those with complete (non-missing) data on the outcome, the socio-demographic exposures and the potential confounding variables listed in Table 1. Standard box-plots the median (middle vertical line of each box) and the 25th and 75th centiles (outside of the box). The whiskers span to up to 1.5 times the interquartile range or the minimum or maximum values. The dots represent outliers beyond this range.

Figure S2. Age-specific trends in breast cancer mortality in Brazil (1979-2013), Norway (1953-2013) and the USA (1950-2013)

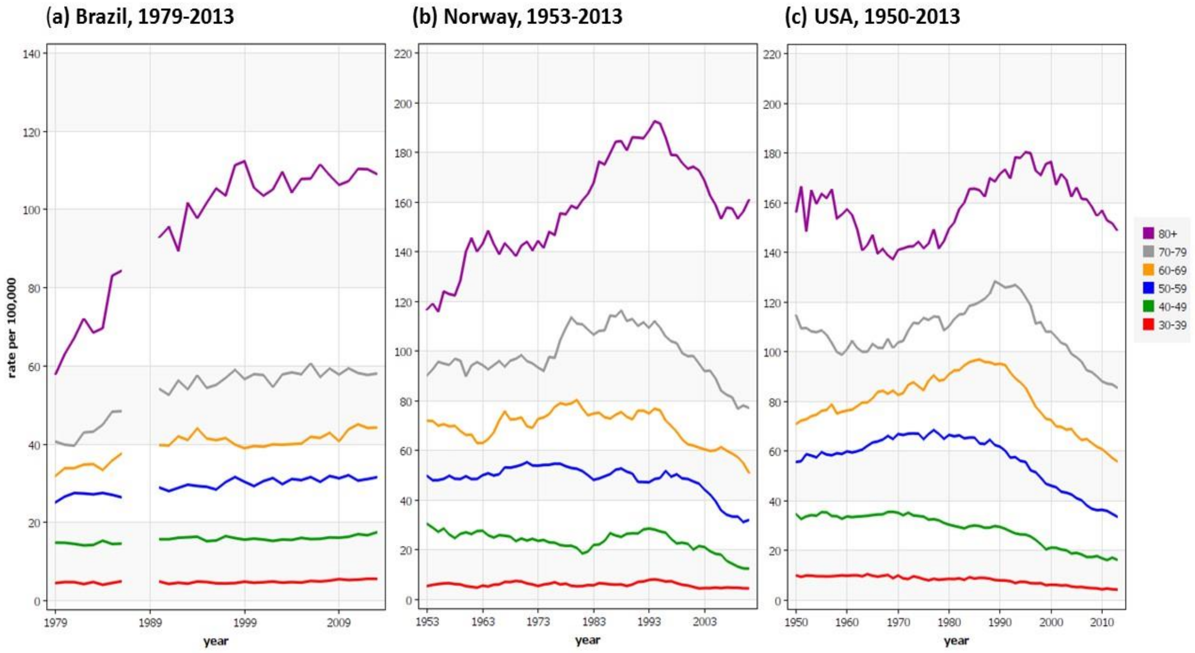

From the International Agency for Research on Cancer (IARC /WHO) Mortality Database (http://www-dep.iarc.fr/WHOdb/WHOdb.htm). Brazilian data for the specific age-groups considered here were not available for the years 1987-1989.
